# Impact of hydroxytyrosol on stroke: tracking therapy response on neuroinflammation and cerebrovascular parameters using PET-MR imaging and on functional outcomes

**DOI:** 10.7150/thno.48110

**Published:** 2021-02-15

**Authors:** Cristina Barca, Maximilian Wiesmann, Jesús Calahorra, Lydia Wachsmuth, Christian Döring, Claudia Foray, Ali Heiradi, Sven Hermann, Maria Ángeles Peinado, Eva Siles, Cornelius Faber, Michael Schäfers, Amanda J Kiliaan, Andreas H. Jacobs, Bastian Zinnhardt

**Affiliations:** 1 European Institute for Molecular Imaging (EIMI), University of Münster, Münster, Germany.; 2 PET Imaging in Drug Design and Development (PET3D).; 3 Department of Medical Imaging, Anatomy, Radboud University Medical Center, Nijmegen, The Netherlands.; 4 Department of Experimental Biology, University of Jaén, Campus las Lagunillas, Jaén, Spain.; 5 Department of Radiology, Translational Research Imaging Center (TRIC), University Hospital Münster, Germany.; 6 Institute of Physiological Chemistry and Pathobiochemistry, University Hospital Münster, Germany.; 7 Immune Image, Innovative Medicines Initiative (IMI).; 8 Department of Nuclear Medicine, University Hospital Münster, Germany.; 9 Department of Geriatrics and Neurology, Johanniter Hospital, Bonn, Germany.; 10 Biomarkers and Translational Technologies, Roche Pharma Research and Early Development, Roche Innovation Center Basel, F. Hoffmann-La Roche Ltd., Basel, Switzerland.

**Keywords:** Hydroxytyrosol, transient middle artery occlusion, neuroinflammation, [^18^]DPA-714, multimodal imaging, TSPO

## Abstract

Immune cells have been implicated in influencing stroke outcomes depending on their temporal dynamics, number, and spatial distribution after ischemia. Depending on their activation status, immune cells can have detrimental and beneficial properties on tissue outcome after stroke, highlighting the need to modulate inflammation towards beneficial and restorative immune responses. Novel dietary therapies may promote modulation of pro- and anti-inflammatory immune cell functions.

Among the dietary interventions inspired by the Mediterranean diet, hydroxytyrosol (HT), the main phenolic component of the extra virgin olive oil (EVOO), has been suggested to have antioxidant and anti-inflammatory properties *in vitro*. However, immunomodulatory effects of HT have not yet been studied *in vivo* after stroke.

The aim of this project is therefore to monitor the therapeutic effect of a HT-enriched diet in an experimental stroke model using non-invasive *in vivo* multimodal imaging, behavioural phenotyping and cross-correlation with *ex vivo* parameters.

**Methods:** A total of N = 22 male C57BL/6 mice were fed with either a standard chow (n = 11) or a HT enriched diet (n = 11) for 35 days, following a 30 min transient middle cerebral artery occlusion (tMCAo). T_2_-weighted (lesion) and perfusion (cerebral blood flow)-/diffusion (cellular density)-weighted MR images were acquired at days 1, 3, 7, 14, 21 and 30 post ischemia. [^18^F]DPA-714 (TSPO, neuroinflammation marker) PET-CT scans were acquired at days 7, 14, 21 and 30 post ischemia. Infarct volume (mm^3^), cerebral blood flow (mL/100g/min), apparent diffusion coefficient (10^-4^·mm^2^/s) and percentage of injected tracer dose (%ID/mL) were assessed.

Behavioural tests (grip test, rotarod, open field, pole test) were performed prior and after ischemia to access therapy effects on sensorimotor functions.

*Ex vivo* analyses (IHC, IF, WB) were performed to quantify TSPO expression, immune cells including microglia/macrophages (Iba-1, F4/80), astrocytes (GFAP) and peripheral markers in serum such as thiobarbituric acid reactive substances (TBARS) and nitric oxide (NO) 35 days post ischemia. Additionally, gene expression of pro- and anti-inflammatory markers were assessed by rt-qPCR, including *tspo*, *cd163*, *arg1*, *tnf* and *Il-1β*.

**Results:** No treatment effect was observed on temporal [^18^F]DPA-714 uptake within the ischemic and contralateral region (two-way RM ANOVA, *p* = 0.71). Quantification of the percentage of TSPO^+^ area by immunoreactivity indicated a slight 2-fold increase in TSPO expression within the infarct region in HT-fed mice at day 35 post ischemia (*p* = 0.011) correlating with a 2-3 fold increase in Iba-1^+^ cell population expressing CD163 as anti-inflammatory marker (R^2^ = 0.80). Most of the GFAP^+^ cells were TSPO^-^. Only few F4/80^+^ cells were observed at day 35 post ischemia in both groups.

No significant treatment effect was observed on global ADC and CBF within the infarct and the contralateral region over time. Behavioural tests indicated improved strength of the forepaws at day 14 post ischemia (*p* = 0.031).

**Conclusion:** An HT-enriched diet significantly increased the number of Iba-1^+^ microglia/macrophages in the post-ischemic area, inducing higher expression of anti-inflammatory markers while no clear-cut effect was observed. Also, HT did not affect recovery of the cerebrovascular parameters, including ADC and CBF.

Altogether, our data indicated that a prolonged dietary intervention with HT, as a single component of the Mediterranean diet, induces molecular changes that may improve stroke outcomes. Therefore, we support the use of the Mediterranean diet as a multicomponent therapy approach after stroke.

## Introduction

Decreased cerebral blood flow (CBF) due to the occlusion of the blood vessels leads to ischemic stroke. Subsequently, activation of pathological mechanisms such as oxidative stress, excitotoxicity, inflammation, and apoptosis, potentiates the ischemic injury. Each of those pathological processes have been the central focus of research for new stroke therapies. However, the treatment options are still limited: acute treatment with the recombinant tissue plasminogen activator to resolve obstructive blood clots is the only FDA-approved drug for acute intervention, apart from mechanical thrombectomy 1,2. However, it has a restrictive time window and may induce severe complications 3 such as haemorrhagic transformation 4. Furthermore, no other drug is available in clinical practice for the rehabilitation of patients at later stages of the disease.

An ischemic event and potential reperfusion of the injured tissue causes a misbalance in the production of free radicals (reactive oxygen (ROS) and nitrogen (RNS) species) in brain parenchyma and periphery 5,6. Efforts have focused on new neuroprotective compounds able to block or scavenge the excess of free radicals 3,5. While animal research supported the use of antioxidant agents, most of these antioxidants fail to be effective in human clinical trials because of the short half-life and/or human comorbidities 3,5.

Oxidative stress contributes to the post-ischemic inflammatory response, with the release of inflammatory mediators and concomitant blood-brain barrier disruption. As a result, an inflammatory environment emerges, driven by activated innate and peripheral immune cells playing a pivotal role in stroke resolution. Although neuroinflammation (NI) is key for tissue repair and remodelling, it may also be detrimental by increasing tissue injury 7. The balance between pro- and anti-inflammatory stimuli drives the functional polarization of many immune cells such as microglia/macrophages (Iba-1^+^, F4/80^+^, CD11b^+^ cells), astrocytes (GFAP^+^ cells) and neutrophils into beneficial or detrimental phenotypes 8,9. In stroke, microglia/macrophages showed an enhanced beneficial anti-inflammatory phenotype within the first week, but expressed a more detrimental pro-inflammatory genes signature at later stages 8,10, peaking around day 14 post ischemia 11. However, the concept of differential waves of anti- and pro-inflammatory microglia is still too simplistic to illustrate the complex spatiotemporal changes of the transcriptomic and proteomic microglial profile in response to injury 12,13.

Although both processes occur sequentially, the anti-inflammatory response might not be sufficient very early after stroke to balance the detrimental effects of the pro-inflammatory burst. The degree and heterogeneity of immune cells in post-ischemic tissues make the inflammatory response a potential drug target for stroke immunotherapy 8,14. Therapeutic strategies include enhancing an anti-inflammatory environment, inhibition of pro-inflammatory stimuli and a phenotypical switch toward an anti-inflammatory phenotype. Despite significant advances towards better therapies, no immunomodulatory drugs are available yet 7,10,14. The future success of immunomodulatory therapies rely on the elucidation of the immune interactions and promote tissue repair 15.

Hydroxytyrosol (3,4-dihydroxyphenyl-ethanol, HT) 16 is recognized as a potent antioxidant, anti-inflammatory, and neuroprotective molecule 17-20. HT is redundantly associated to the Mediterranean diet since it is the main phenolic compound of the extra virgin olive oil (EVOO) extracts 20-24. Human trials indicated that an EVOO-based Mediterranean diet reduced the risk of stroke 25,26, raising interest as a complementary beneficial approach. HT exerts a wide range of beneficial effects while mechanisms of action are still unclear 17. HT elicits direct scavenging effects on ROS/RNS but also activates mechanisms that counteract oxidative damage 6,17,24, by protecting viable tissue and vascular cells 24,27. Concomitant with its strong antioxidant property, HT has been reported to possess high anti-inflammatory activity *in vitro* and in animal models of inflammation 17,28,29, by inhibiting the production of pro-inflammatory markers 19,29. *In vitro* evidences showed decreased cyclooxygenase-2 (COX-2), inducible nitric oxide synthase (iNOS), and MMP-9 levels in activated murine macrophages 30, and stimulated human monocytes 31, mediated by inhibition of the nuclear factor-kB (NF-kB) and PKCα/PKCβ1 respectively. However, contradictory results in human macrophages were obtained by Bigali et al. (2018), showing no effect on iNOS but on prostaglandin E2 (PGE2) levels and transcription factor Nrf2, overall attenuating inflammation 32. An *in vivo* study confirmed the reduction of COX-2 and tumor necrosis factor alpha (TNF-α) levels in plasma in a murine inflammation model 29.

Despite its role in preventing ischemic events 26,33, the therapeutic effects of HT have been scarcely studied in stroke animal models 34.

In the present study, we investigated the possible therapeutic effects of a HT-enriched diet as therapeutic intervention after transient ischemic stroke on the neuro-inflammatory response, cerebrovascular, and functional parameters in a transient middle cerebral artery occlusion (tMCAo) mouse model (30 minutes) using *in vivo* multimodal imaging and behavioural tests. To do so, we performed a longitudinal positron emission tomography (PET)/magnetic resonance (MR) imaging study to monitor the underlying mechanisms occurring at the different stages of the disease and therapy response. We investigated the effects of HT on the TSPO-dependent neuroinflammatory reaction, the infarct volume and vascular parameters (diffusion and perfusion). We also assessed sensorimotor functions prior and after surgery and therapy effect on motor functions recovery using behavioural tests. Blood samples were collected on the last day of the experiment to assess peripheral markers including nitric oxide (NO) and thiobarbituric acid reactive substances (TBARS), as indexes of vascular integrity and lipid peroxidation, respectively. Brains were collected on the last day of the experiment to investigate the cellular source of TSPO expression in the chronic phase, including microglia/macrophages (Iba-1^+^, F4/80^+^), expression of the anti-inflammatory marker CD163 and astrocytes (GFAP^+^).

We hypothesized that HT may have immunomodulatory functions in the post-ischemic phase, able to modify the inflammatory landscape. Potential therapy effects may be non-invasively detected by [^18^F]DPA-714 as a biomarker for glial activation and by T_2_-weighted MR (lesion), diffusion-weighted (cellular density) and perfusion-weighted (cerebral blood flow) MR imaging. Additionally, beneficial functional outcomes may be detected by behavioural tests including open field (locomotion), pole test (general motor functions), grip strength test (strength) and rotarod (coordination).

## Methods

### Animals

All experiments were conducted in accordance with the German Law on the Care and Use of Laboratory Animals and approved by the Landesamt für Natur, Umwelt und Verbraucherschutz of North Rhine-Westphalia and according to the ARRIVE guidelines (https://www.nc3rs.org.uk/arrive-guidelines) 35.

3-4 months old C57BL/6J mice (N = 22, 20-25 g in body weight) were housed in a temperature-controlled (25 °C) and humidity-controlled (40%) facility with a 12:12h light:dark cycle. Food and water were available ad libitum.

After transient MCA occlusion, mice were randomly assigned by an external person to either a control (n = 11) or a hydroxytyrosol (n = 11) diet. Experimenters were blinded during the procedures and did not know which animal was assigned to the treatment group until the data analysis was performed.

**Figure 1:**
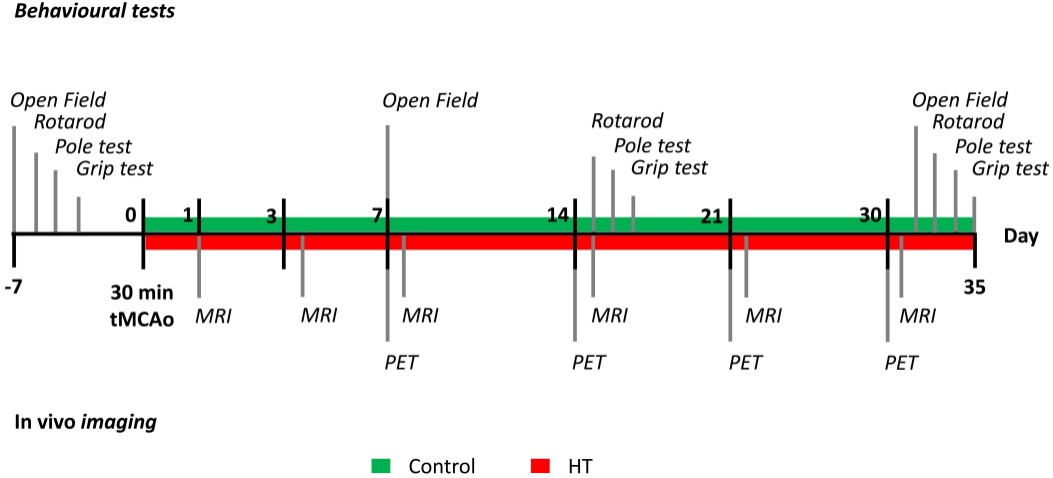
** Study design.** After a 30 minutes tMCAo, N = 22 mice were randomly divided into a control (green) or HT (red) diet group. MR studies were conducted at days 1, 3, 7, 14, 21 and 30 post ischemia while [^18^F]DPA-714 PET scans were acquired at days 7, 14, 21 and 30 post ischemia. Mice were also tested for motor and cognitive impairments using four behavioural tests: open field, grip strength test, pole test, and rotarod prior and after a 30 min tMCAo. After the last behavioural test (at day 35), all animals were killed and brains were collected for *ex vivo* analysis.

N= 8 mice per group were used for *in vivo* imaging purpose and behavioural assessment. Besides, n = 3 mice per group were added for *ex vivo* analysis at day 35 post ischemia. Animals were anesthetized, sacrificed by transcardiac perfusion after completion of the study, at day 35 post ischemia. Brains were removed for further *ex vivo* analyses. The timeline of the experiment design is summarized in Figure 1. Additional details on the study design and animal number were reported in Supplementary Table S1.

### Transient middle cerebral artery occlusion (tMCAo) and reperfusion

A total of N = 22 male C57BL/6J mice, 3-4 months, old were subjected to a 30 min transient middle cerebral artery occlusion (tMCAo) with partial reperfusion at day 0 (Figure 1, Figure S1), as previously described with minor changes 36,37. Briefly, animals received an i.p. injection of 0.04 mg Fentanyl/4 mg Midazolam/1 g bodyweight (Fentanyl: RotexMedica, Trittau, Germany; Midazolam: Ratiopharm, Ulm, Germany) and were anaesthetised with 4% v/v Isoflurane/O_2_ (Abbott Animal Health) and maintained with 2% v/v Isoflurane/O_2_. Body temperature was maintained at 37 °C with a heating plate system. The regional cerebral blood flow (CBF) was monitored by laser Doppler flowmetry (Periflux System 5000, Perimed, Järfälla, Sweden) during the entire procedure to ensure successful occlusion and reperfusion (Figure S1). To do so, the laser Doppler probe (PH07-5, Perimed, Järfälla, Sweden) was directly fixed on the skull above the cortical area perfused by the middle cerebral artery (MCA) and the signal was recorded with PeriSoft software (Perimed, Järfälla, Sweden).

Middle cerebral artery occlusion was achieved by introducing a silicone-coated 7-0 MCAO monofilament (diameter with coating 0.19 ± 0.01 mm) (Doccol Corporation, MA, USA) into the distal common carotid artery (CCA), following then the internal carotid artery upwards to the middle cerebral artery. Success occlusion induced the Doppler signal to drop by at least 80 % of the baseline signal followed by partial reperfusion. The monofilament was fixed for 30 minutes before withdrawal. Right after surgery, all animals received subcutaneously Buprenorphin (0.05-0.1 mg/kg bodyweight) (Indivior, Berkshire, UK) for pain relief. Mice were controlled and weighted daily during the first week, and then once a week until the end of the experiment. Exclusion criteria were: (i) absence of lesion on the T_2_w-weighted MR images on day 1 post ischemia, (ii) edema volume exceeding the cortical and striatal tissues, and (iii) extreme weight loss (> 20% of the initial weight).

### Hydroxytyrosol diet

The hydroxytyrosol diet contained 0.03 gm% hydroxytyrosol resulting in an estimated daily HT intake of 45 mg/kg body weight daily HT intake (Seprox Biotech, Murcia, Spain). Calahorra et al. (2019) published individual food intake of the HT diet and change in body weight after stroke 34. Food intake decreased within the first week post ischemia but increased again afterwards.

Composition of both control and HT diets included 24.0% kcal protein, 15.0% kcal fat and 61.0% kcal carbohydrates (Research Diet Services B.V., Wijk bij Duurstede, The Netherlands), as previously reported 34,38,39.

Diets were stored at -20 °C until use.

### Behavioural testing

Open field, grip test, rotarod, and pole tests were performed to evaluate the effects of a HT-enriched diet on motor and cognitive dysfunctions after ischemia. The four behavioural tests were carried out prior to tMCAo and at days 7, 14 and 30 post ischemia as indicated in Figure 1.

### Open field

The open field test allowed for the assessment of behaviour and locomotor activity by recording free movements of a mouse. The open field maze consists of a squared chamber (40 cm (length) × 40 cm (width) × 25 cm (height)) made of non-porous plastic placed in a quiet and free of clue environment. The mouse is placed in the middle of the maze and can freely move for 10 min. Locomotion was automatically recorded using EthoVision XT15 (Noldus, Wageningen, The Netherlands) video tracking system attached to a pole above the field.

### Grip strength test

The grip strength test allows assessing grip strength of a mouse using a grip strength meter (Grip-Strength Meter, 47200, Ugo Basile, Italy) to determine forelimb and also total limb (fore- and hindlimbs combined) muscle strength.

The grip strength meter is arranged horizontally on the table. The mouse is able to grasp the grip trapeze with the forepaws or the grid with both fore- and hind paws while the experimenter holds the tail. The mice are pulled backwards until the grip is released. The test is repeated five consecutive times. The average of the peak force (in gf) is calculated from the last three trials.

### Rotarod

The rotarod (ITC LifeScience Inc., Woodland Hills, CA, USA) test is used to evaluate the motor coordination of rodents. In this test, a mouse is placed on a rotating rod (3 cm in diameter) suspended 30 cm above the protected-rotarod apparatus floor. The mouse is placed on the rod and left to acclimatize for 30 s. Then, the rotarod is turned on to accelerate in 300 s from 4 to 40 rpm. The trial is complete when the mouse falls down. The test is repeated five consecutive times. The time spent on the cylinder (in seconds) and the covered distance (in centimetres) were automatically recorded for each trial. The average values are calculated from the last three trials. Two-way repeated measures ANOVA indicated no treatment, time, or interaction significant effect on run distance. Results are reported in Figure S11.

### Pole test

The pole test is a general test to assess motor functions. The mouse is placed head upward just below the top of a vertical pole (diameter 2.5 cm; height 60 cm) and then allowed to descend into their home cage. Each trial is recorded using EthoVision XT15 (Noldus, Wageningen, The Netherlands) to manually measure the time needed to reach the floor. This procedure is repeated five times but only the last three trials are used for calculation.

Two-way repeated measures ANOVA indicated no treatment, time or interaction significant effect on run distance. Results are reported in Figure S12.

### [^18^F]DPA-714 PET imaging

[^18^F]DPA-714 was prepared as previously described 40 with a > 99% radiochemical purity. [^18^F]DPA-714 PET scans were acquired on a high-resolution small animal PET scanner (32 module quadHIDAC, Oxford Positron Systems Ltd.).

Once anaesthetized (induction: 5% v/v isoflurane/O_2_), mice were cannulated using a 26G catheter (Vasculon Plus, BD, Heidelberg, Germany) connected to polyethylene tube filled with NaCl. Then, mice were injected 12 ± 3.9 MBq of [^18^F]DPA-714 tracer and kept under anaesthesia (1.5% v/v isoflurane/O_2_) in a warmed environment for 45 minutes. The PET scan was acquired from 45 to 60 minutes post injection.

PET data were reconstructed using one-pass list mode expectation maximization algorithm with resolution recovery 41,42, allowing reconstruction of a large number of coincidence events into a large field of view (FOV). Spatial resolution is of 1.07 mm with uniform resolution over the entire FOV (280 mm axial length, 165 mm diameter). After the PET scan, the animal bed was transferred in the computed tomography CT scanner (Inveon, Siemens Medical Solutions) for anatomic co-registration with the PET image. Three molecular sieve spheres (Acros Organics, Geel, Belgium) were rinsed in radiotracer solution and taped on the extremities of the bed (two on the left side and one on the right side). Those landmarks are both visible on PET and CT images and used for co-registration.

### Magnetic resonance imaging

Magnetic resonance imaging (MRI) scans (Biospec 94/20, Bruker Biospin GmbH, Ettlingen, Germany), including T_2_-weighted (T_2_w), diffusion weighted imaging (DWI) and perfusion weighted imaging (PWI), were conducted at days 1, 3, 7, 14, 21 and 30 after tMCAo. Infarct volume, quantitative apparent diffusion coefficient (ADC) and cerebral blood flow were calculated. Further experimental details can be found in Supplementary Material and Methods.

### Image analysis

The full *in vivo* imaging dataset was analysed using the in-house developed software *MEDgical* dedicated to the analysis of multi-dimensional, multi-scale biomedical image data.

First, all MR images were manually co-registered to the corresponding PET-CT scans acquired on the same day from the same animal. Then, T_2_w-MR images from day 1 post ischemia were co-registered with all the PET-CT scans to visualize spatio-temporal distribution of [^18^F]DPA-714 tracer and quantify the regional uptake.

Besides, co-registered T_2_w-MR images were segmented using a modified atlas-based thresholding approach 43 to delineate the infarcted region at days 1, 3 and 7 post ischemia. Briefly, an atlas-based right hemisphere volume of interest (VOI) was segmented using a threshold of the mean intensity + 2.5 * standard deviation (sd) of the atlas-based contralateral striatum.

The percentage of radiotracer uptake per millilitre (%ID/mL) was assessed for all time points using the infarct volume defined on the T_2_w-MR image from day 1 and the atlas-based contralateral striatum VOI (previously adjusted to the T_2_w-MR images).

Apparent diffusion coefficient (ADC) maps and arterial spin labelling (ASL) maps were co-registered with their respective T_2_w-MR images using our in-house developed software. ADC and CBF values were assessed within (i) the infarct defined by the T_2_w-MR image from day 1 post ischemia and (ii) its mirrored VOI into the contralateral hemisphere. Qualitative and quantitative assessments were cross-validated using the original data files (2dseq files) opened with ImageJ 1.51j software (National Institutes of Health, Bethesda, MD, USA). From there, infarct-to-contralateral ratios were calculated for both ADC and CBF values (Figures shown in Supplementary data).

### Tissue processing

On the last day of the experiment, half of the mice were sacrificed by transcardiac perfusion using 0.1 M phosphate-buffered saline (PBS) followed by 4% paraformaldehyde (PFA) in 0.1 M PBS) solution (pH = 7.4, at 4 °C). The brains were harvested and processed for immunohistochemical and immunofluorescent staining.

The other half were only perfused with 0.1 M PBS solution. Brains were immediately frozen in liquid nitrogen and kept at -80 °C for western blotting.

Blood samples were collected before sacrifice and centrifuged (4 °C). Supernatant and pellet were stored at -80 °C until use.

### Plasma analysis

Serum nitric oxide (NO) concentration was determined using an ozone chemiluminescence-based assay as previously described 34,44. Briefly, samples were deproteinized (in 0.8 N NaOH and 16% ZnSO_4_ buffers) and used for chemiluminescence assay in a NO analyser (NOA 280i; Sievers Instruments, Boulder, CO, USA).

Serum thiobarbituric acid reactive substances (TBARS) levels were assessed by an adapted method previously described 34,45.

Analyses indicated no treatment effects on NO nor TBARS. Results are reported in Figure S10.

### Immunoreactivity

5 μm paraffin sections were cut from mouse brains fixated in 4% paraformaldehyde and subsequently immunohistochemically stained and analysed.

Primary antibodies and dilutions used: anti-PBR antibody (anti-PBR, TSPO) (rabbit, 1:250, ab109497, Abcam, Cambridge, UK), anti-ionized calcium binding adapter molecule 1 (anti-Iba-1) (rabbit, 1:500, 019-19741, Wako Chemicals USA, Inc. Richmond, VA, USA), anti-glial fibrillary acidic protein (anti-GFAP) (chicken, 1:500, ab4675, Abcam, Cambridge, UK), anti-F4/80 (rat, 1:200, ab6640, Abcam, Cambridge, UK) and CD163 (rabbit, 1:50, bs-2527R, Bioss). Tissue sections were investigated with a combined fluorescent-light microscope (Nikon Eclipse NI-E, Nikon, Tokyo, Japan). Details are given in Supplementary Material and Methods.

### Western blot

Western blot was performed to determine protein levels in the infarcted and non-infarcted hemispheres. Proteins were extracted in a radio-immunoprecipitation buffer (RIPA). A total of 30 g of total proteins were separated in a 4%-20% gradient gel (Bio-Rad Laboratories, Hercules, CA, USA). Proteins were then transferred to a polyvinylidene fluoride (PVDF) membrane. The membranes were blocked and incubated overnight at 4 °C with the primary anti-PBR antibody (anti-PBR, TSPO) (1:500, ab109497, Abcam, Cambridge, UK). Besides, GAPDH was used to normalize protein loading and transfer (ab37168, Abcam, Cambridge, UK). The membranes were then incubated with the corresponding secondary antibody and blots were developed by chemiluminescence. The intensity of bands was quantified using ImageJ 1.51j software (National Institutes of Health, Bethesda, MD, USA). Normalized band intensities were reported as relative protein expression.

### Real time qPCR

Total RNA isolated from control and HT-fed mice half-brain were kindly provided by A. J. Kiliaan's working group. We processed the samples with DNAse I treatment (Roche, Mannheim, Germany) to avoid contaminations from genomic DNA. The isolated RNA was quantified (Nanodrop, Thermo Scientific). One µg of total RNA was reverse transcribed into first-strand cDNA using the Transcriptor First Strand cDNA Synthesis Kit (Roche, Indianapolis, IN).

The forward (FW) and reverse (RV) primer sequences (Sigma-Aldrich) used were: tspo (FW, ggatctttccagaacatcag; RV, acgtacaaagtaggctcc), Il1b (FW, ggatgatgatgataacctgc; RV, catggagaatatcacttgttgg Arg1 (FW, ctgacctatgtgtcatttgg; RV, catctgggaactttcctttc), Gapdh (FW, ctggagaaacctgccaagta; RV, tgttgctgtagccgtattca), Tnf (FW, tgagactgagatcta, RV, ctagggtacgatcgatagc) and Cd163 (FW, agtctgctcacgatacatag, RV, tccttctggaatagattggg).

Rt-qPCR was performed using the Rotor-Gene SYBR Green master mix with the Rotor-Gene Q device (Qiagen). Relative gene expression changes were assessed using the ∆∆Ct method, with Gapdh as a housekeeping gene.

### Statistical analysis

All statistical analyses were performed using SigmaPlot (Systat Software GmbH, Erkrath, Germany). All data were tested for normality and equal variance using the Shapiro-Wilk and Brown-Forsythe tests, respectively.

The sample sizes were calculated a priori during the animal ethics dossier application. They were determined based on effect size (*p* = 0.05, statistical power: 0.80), mortality rates, and a previous stroke study 46, where we investigated the therapeutic effect of a dietary approach on brain inflammation assessed by [^18^F]DPA-714 PET imaging study in ischemic mice. We set the minimal detectable difference in means to 0.2 and the expected standard deviation of residuals to 0.1.

Peripheral markers data were analysed using unpaired t-tests for two-group comparisons. Longitudinal tracking of stroke-associated parameters ([^18^F]DPA-714 uptake, CBF, ADC, behaviour, etc.) were analysed using two-way ANOVA followed by Holm Sidak's post hoc test for multiple comparisons.

In all statistical tests, differences were considered when *p* < 0.05. Data are expressed and depicted as means ± sd if not specified.

## Results

### T_2_-weighted MRI-defined edema is not affected by HT

First, we aimed at evaluating the impact of a HT diet on the T_2_w-MRI-defined edema volume delineated using a semi-automated atlas-based thresholding method as described by Mulder et al. (2017) 43. This analysis method was established within the first week post ischemia and thus, we only reported quantification of T_2_w-MRI lesion size within the first week. The dataset passed the Shapiro-Wilk (*p* = 0.44) and Brown-Forsythe (*p* = 0.25) tests. Two-way RM ANOVA indicated main effect of time (*p* < 0.001) but not of treatment (*p* = 0.82) or interaction (*p* = 0.95).

For all stroke mice, edema decreased over time. Edema volumes on day 3 (control: *p* = 0.01, HT: *p* = 0.005) and 7 (*p* < 0.001 for both groups) were significantly reduced compared to day 1 post ischemia for both control and HT-fed mice. Similarly, T_2_w-MRI-defined edema volumes on day 7 (*p* < 0.001 for both groups) were significantly smaller compared to day 3 post ischemia (Sidak's post hoc test).

**Figure 2:**
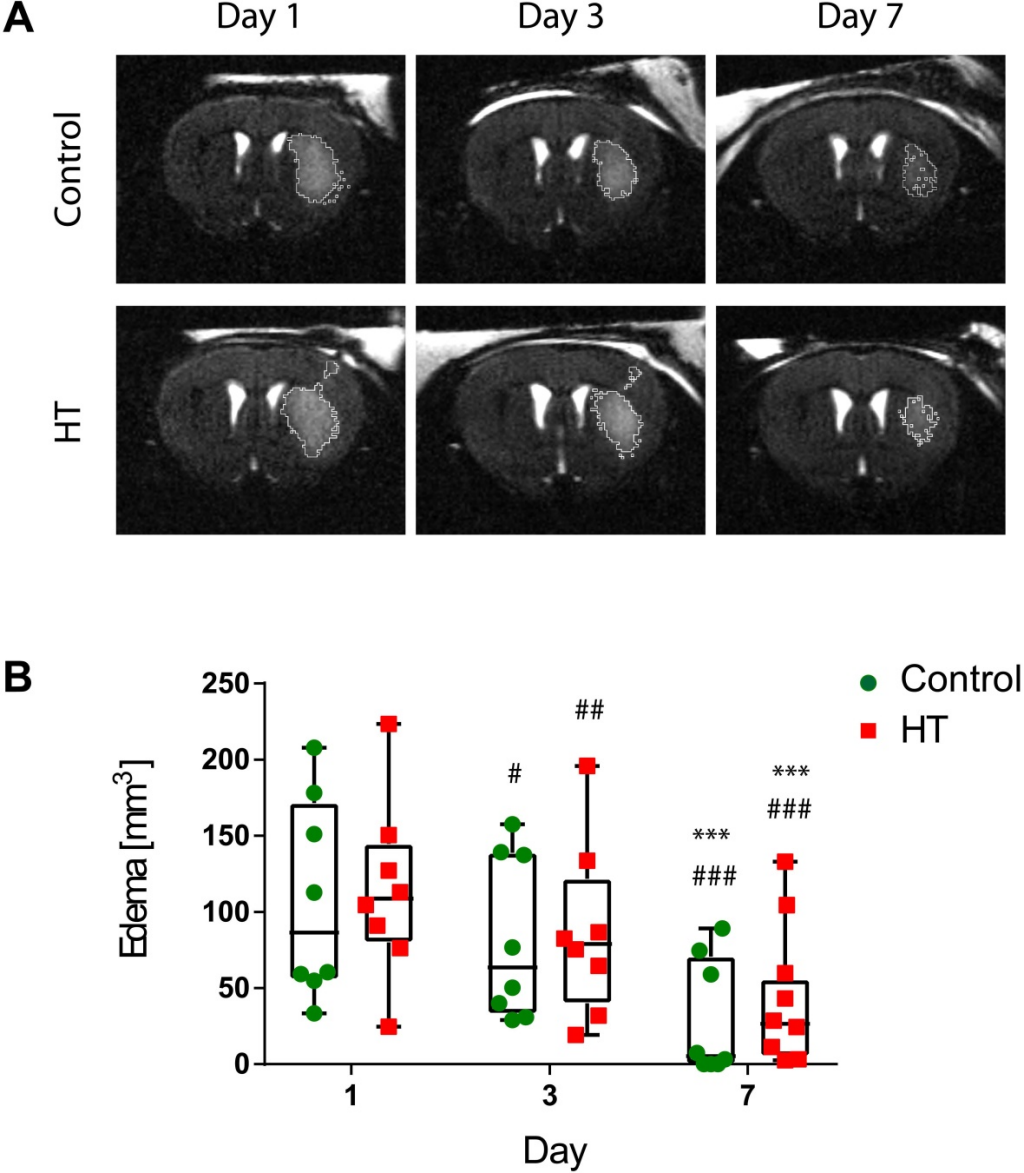
** Longitudinal monitoring of the edema volume using T_2_w-MR imaging after ischemic stroke.** (A) Representative T_2_w-MR images from control and HT-fed mice acquired at days 1, 3 and 7 post ischemia. The edema volume (white) was delineated by an atlas-based thresholding approach. (B) Edema volume decreased from day 3 post ischemia for either control (green, n = 8) and HT (red, n = 8) fed mice. No treatment effect was observed on edema volume. Individual data are plotted in mm^3^. Statistical analysis was carried out with two-way RM ANOVA followed by Holm Sidak's post hoc test for multiple comparisons (* *p* < 0.05, ** *p* < 0.01, *** *p* < 0.005; * *vs.* day 3, # *vs*. day 7).

No treatment effect was observed on the mean T_2_w-MR edema volume for each time point (Figure 2). Importantly, edema volumes on day 1 were comparable between the two groups (Sidak's post hoc test, *p* = 0.80).

Individual follow-ups of the T_2_w-MRI edema are shown in Supplementary Figure S2.

### Hydroxytyrosol does not improve reperfusion of the infarct

**Figure 3:**
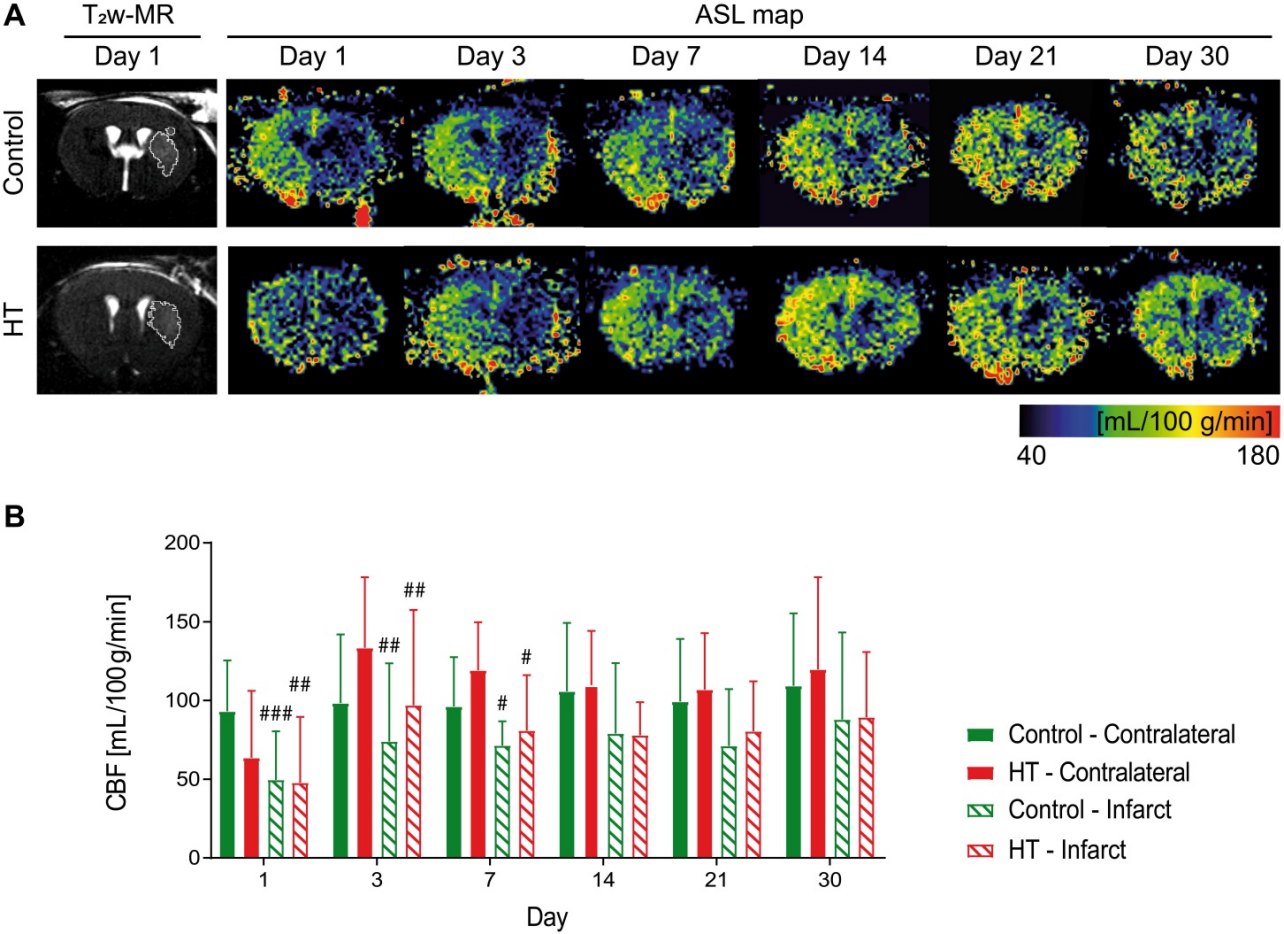
** Longitudinal monitoring of the mean cerebral blood flow by FAIR-MRI 1, 3, 7, 14, 21 and 30 days after stroke.** CBF was assessed within the infarct defined on the T_2_w-MR images from day 1 post ischemia and this mirrored ROI in the contralateral side in control (green) and HT-fed (red) mice. (A) Representative CBF images at days 1, 3, 7, 14, 21 and 30 post ischemia for both control (upper row) and HT-fed mice (lower row) and the corresponding T_2_w-MR image at day 1 post ischemia. CBF maps showed hypoperfused infarct regions within the first week for both groups. At later time points, CBF was partially recovered in both groups. (B) Quantification of the mean CBF within the infarct and contralateral striatum showed no treatment effect on tissue reperfusion. Values represent mean ± sd (n = 8 per group). Statistical analysis was carried out with RM ANOVA followed by Holm Sidak's post hoc test for multiple comparisons (# *p* < 0.05, ## *p* < 0.01, ### *p* < 0.005, # *vs.* contralateral).

We tracked CBF changes associated to an ischemic injury and assessed the effect of a HT diet at days 1, 3, 7, 14, 21 and 30 post ischemia using FAIR-RARE MRI (Figure 3A). Mean CBF and ratios dataset passed the Shapiro-Wilk and Brown-Forsythe tests.

Repeated measures ANOVA did not indicate any main effect of time (*p* = 0.21) or treatment (*p* = 0.88) or interaction (*p* = 0.61) on mean CBF values. Post hoc test was not performed for this dataset. In overall, the mean CBF values of both control and HT mice did not significantly change over time, in both infarct and contralateral side.

Longitudinal intra-group comparison indicated that CBF values within the infarct at days 1, 3 and 7 were decreased compared to the corresponding area in the unaffected hemisphere in all stroke mice (RM ANOVA, time, *p* = 0.001). Lower CBF values within the infarct of control mice were observed at days 1 (infarct: 49.7 ± 30.7 mL/100 g/min, contralateral: 103.4 ± 14.8 mL/100 g/min, Sidak's post hoc test, *p* = 0.003) until day 7 (infarct: 71.4 ± 15.2 mL/100 g/min, contralateral: 96.3 ± 28.6 mL/100 g/min, Sidak's post hoc test, *p* = 0.033) compared to the contralateral region. Similarly, mean CBF values within the infarct of HT-fed mice at day 1 (infarct 47.8 ± 41.8 mL/100 g/min; contralateral: 63.5 ± 32.5 mL/100 g/min) until day 7 post ischemia (infarct: 80.8 ± 35.3 mL/100 g/min, contralateral: 119.2 ± 30.5 mL/100 g/min, Sidak's post hoc test, *p* = 0.049) were significantly lower than in the contralateral hemisphere (Figure 3B).

To reduce intra-individual variability, we also assessed the infarct-to-contralateral ratio at each time point. Infarct-to-contralateral CBF ratios dataset passed the Shapiro-Wilk (*p* = 0.35) and Brown-Forsythe (*p* = 0.93) tests. Two-way RM ANOVA indicated main effect of time (*p* < 0.001) but not of the treatment (*p* = 0.43) or interaction (*p* = 0.69). Data are shown in Figure S3.

### Hydroxytyrosol does not affect the water diffusion coefficient

**Figure 4:**
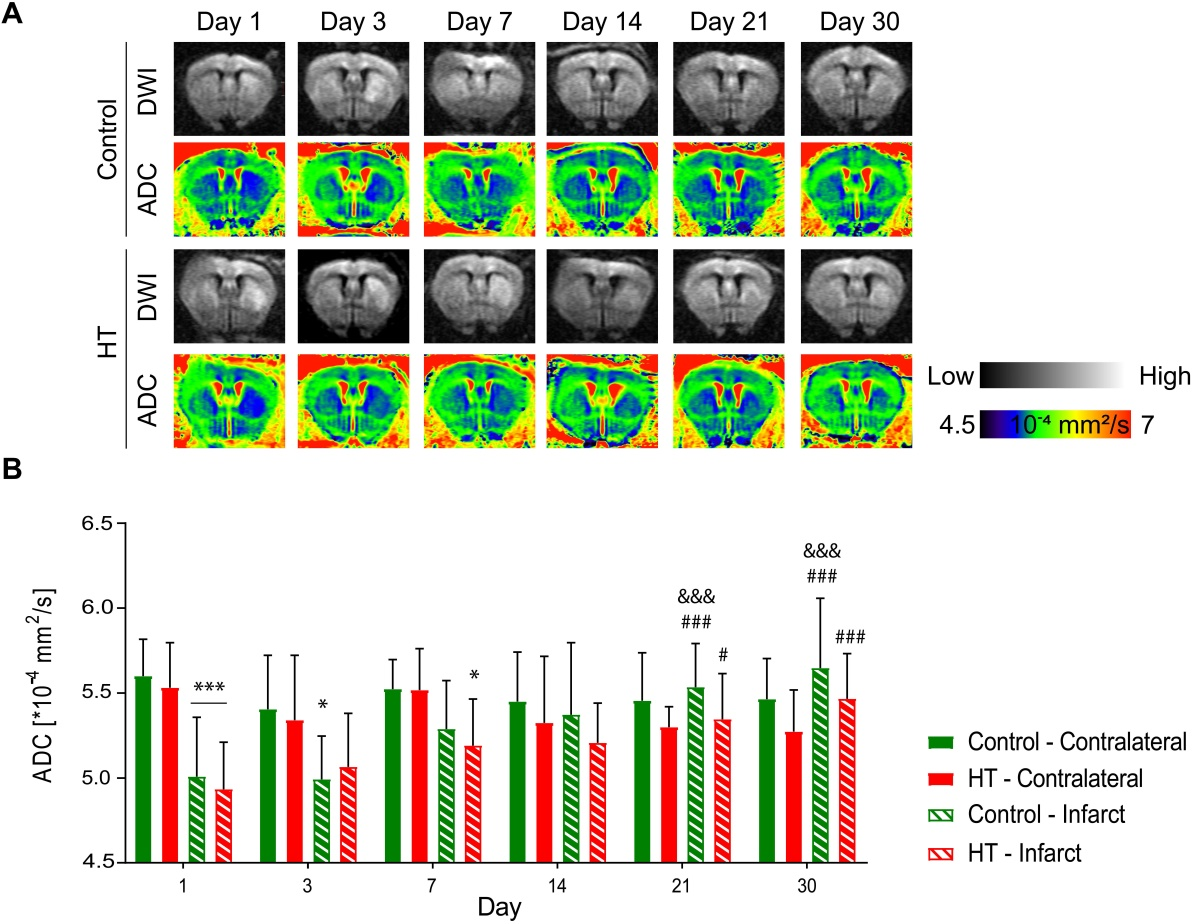
** Hydroxytyrosol does not affect the microstructure after stroke.** Apparent diffusion coefficient was assessed within the infarct defined on the T_2_w-MR images from day 1 post ischemia and a mirrored reference ROI (contralateral) in control (green) and HT-fed (red) mice. (A) Representative diffusion-weighted images (with the highest b value of 2400 s/mm^2^) and the respective ADC map at days 1, 3, 7, 14, 21 and 30 days post ischemia for each experimental group. (B) Time course of the ADC values determined in the infarct and the contralateral area in control (green) and HT-fed (red) mice. RM ANOVA indicated main effect of time (*p* < 0.001) and time * region (*p* < 0.001) but not of treatment (*p* = 0.34). For both groups, the mean ADC value within the infarct hemisphere significantly recovered from day 14 post ischemia. Values represent mean ± sd. Control: n = 8, HT: n = 8. Statistical analysis was carried out with RM ANOVA followed by Holm Sidak's post hoc test for multiple comparisons (*p < 0.05, **p < 0.01, ***p < 0.005; * *vs.* contralateral, # *vs.* day 1, & *vs.* day 3 and + *vs.* day 7).

Apparent diffusion coefficients (ADCs) were assessed by DTI EPI MRI from a series of echo planar imaging (EPI)-images for each dietary group at days 1, 3, 7, 14, 21 and 30 post ischemia (Figure 4A). The ADC dataset passed the Shapiro-Wilk (*p* = 0.11) and Brown-Forsythe (*p* = 0.24) tests. Repeated measures ANOVA indicated main effect of time (*p* < 0.001) and time * region (*p* < 0.001) but not of treatment (*p* = 0.34).

In both experimental groups, the mean ADC value in the contralateral hemisphere did not change over time (*p* = 0.062, Sidak's post hoc test) (Figure 4B). In the infarct hemisphere, time effect was observed in both groups (*p* < 0.001, Sidak's post hoc): ADC value was significantly increased at days 14 (*p* = 0.009), 21 (*p* < 0.001) and 30 (*p* < 0.001) post ischemia compared to day 1 post ischemia. Moreover, ADC values significantly increased from day 3 to day 21 (*p* = 0.001) and 30 (*p* < 0.001) post ischemia.

Similarly, temporal dynamic of the infarct-to-contralateral ADC ratio indicated main effect of time (*p* < 0.001) but not of the treatment (*p* = 0.81) or interaction (*p* = 0.82) (Figure S4). Infarct-to-contralateral ratio on day 1 post ischemia was significantly increased compared to days 14 (*p* < 0.001), 21 (*p* < 0.001) and 30 (*p* < 0.001) (Sidak's post hoc test). Besides, ADC ratios at days 3 and 7 were significantly decreased compared to day 30 and/or day 14 post ischemia, indicating that the ADC ratio recovered within days 7 and 14 post ischemia.

### Hydroxytyrosol does not affect TSPO expression as assessed by [^18^F]DPA-714 PET imaging

**Figure 5:**
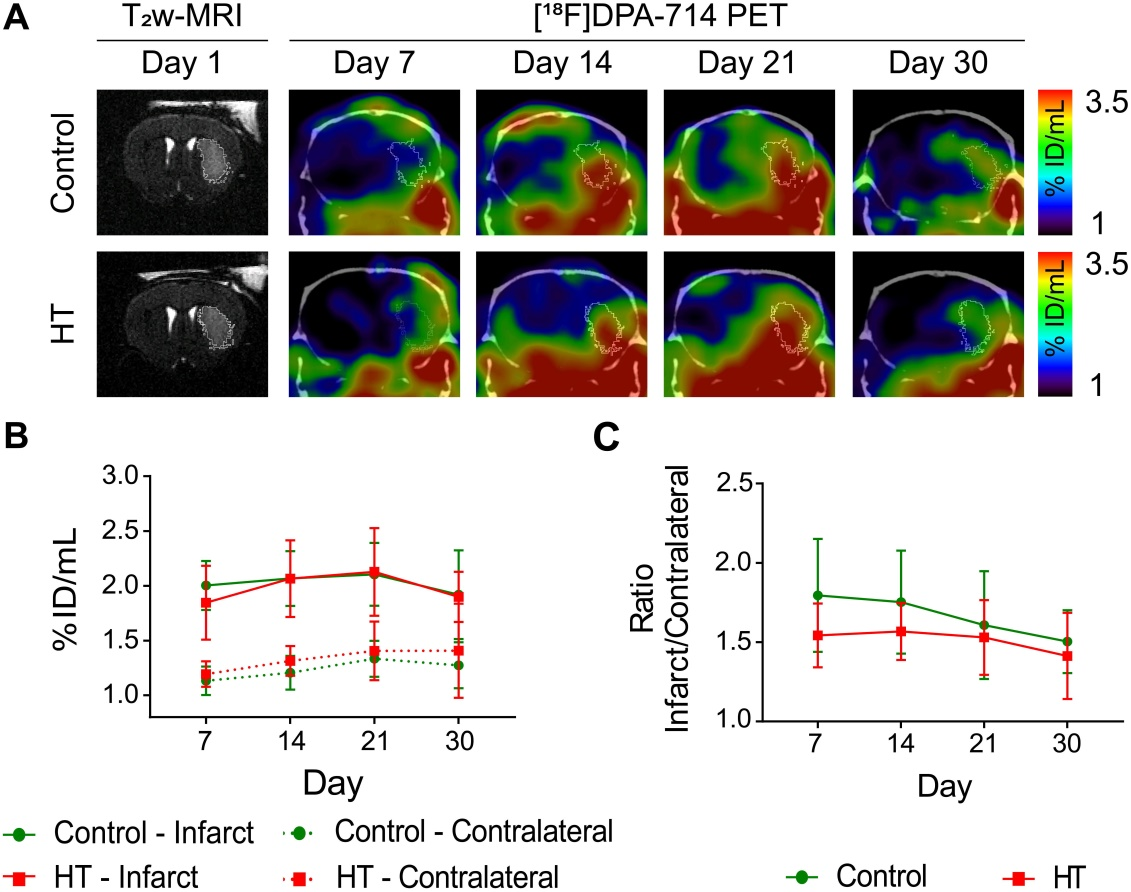
** A HT diet does not affect TSPO expression as detected by [^18^F]DPA-714 PET imaging after ischemic injury.** The mice were scanned for [^18^F]DPA-714 PET at days 7, 14, 21 and 30 post ischemia to assess TSPO expression within the infarct (white circle) and the contralateral striatum. The percentage of injected dose per millimetre (%ID/mL) was quantified within both regions. (A) Representative axial co-registered [^18^F]DPA-714-PET-CT images of a mouse fed with either control (upper row) or HT (lower row) diet after a 30 min tMCAo with comparable T_2_w-infarct on day 1 post ischemia (white delineation). (B) Similar uptake was measured between both groups either within the infarct or contralateral striatum over time. (C) Infarct-to-contralateral ratios did not differ over time, indicative of constant chronic neuroinflammatory reaction in the infarcted hemisphere compared to the contralateral side (*p* > 0.05). Values represent mean ± sd. Control: n = 8, HT: n = 8. Statistical analysis was carried out with two-way RM ANOVA.

[^18^F]DPA-714 uptake dataset passed the Shapiro-Wilk (*p* = 0.70) and Brown-Forsythe (*p* = 0.51) tests. Two-way RM ANOVA indicated no main effect of treatment, time and interaction on tracer uptake within the infarct (Figure 5A-B), and in the contralateral striatum (Figure 5A-B), suggesting that both control and HT-fed mice showed same tracer uptake dynamic over time. Similarly, relative changes in [^18^F]DPA-714 uptake did not indicate any significant difference between treatment groups.

Elevated [^18^F]DPA-714 uptake was observed within and beyond the ischemic lesion defined by T_2_w-MR image on day 1 post ischemia (Figure 5A). Significant difference in tracer uptake was observed between the infarct and contralateral striatum over time, for both treatment groups (Figure 5B). Two-way RM ANOVA revealed a significant effect of the brain region (*p* < 0.001) but not of time (control: *p* = 0.36; HT: *p* = 0.15) or interaction region * time (control: *p* = 0.59; HT: *p* = 0.71). Details are given in the Figure S5.

Infarct-to-contralateral striatum uptake ratios did not differ between control and HT-fed mice over time (two-way RM ANOVA followed by Sidak's post hoc test, treatment: *p* = 0.13, time: *p* = 0.11, treatment * time: *p* = 0.75) (Figure 5C).

### Cross-validation of [^18^F]DPA-714 PET with TSPO expression at day 35 post ischemia

**Figure 6:**
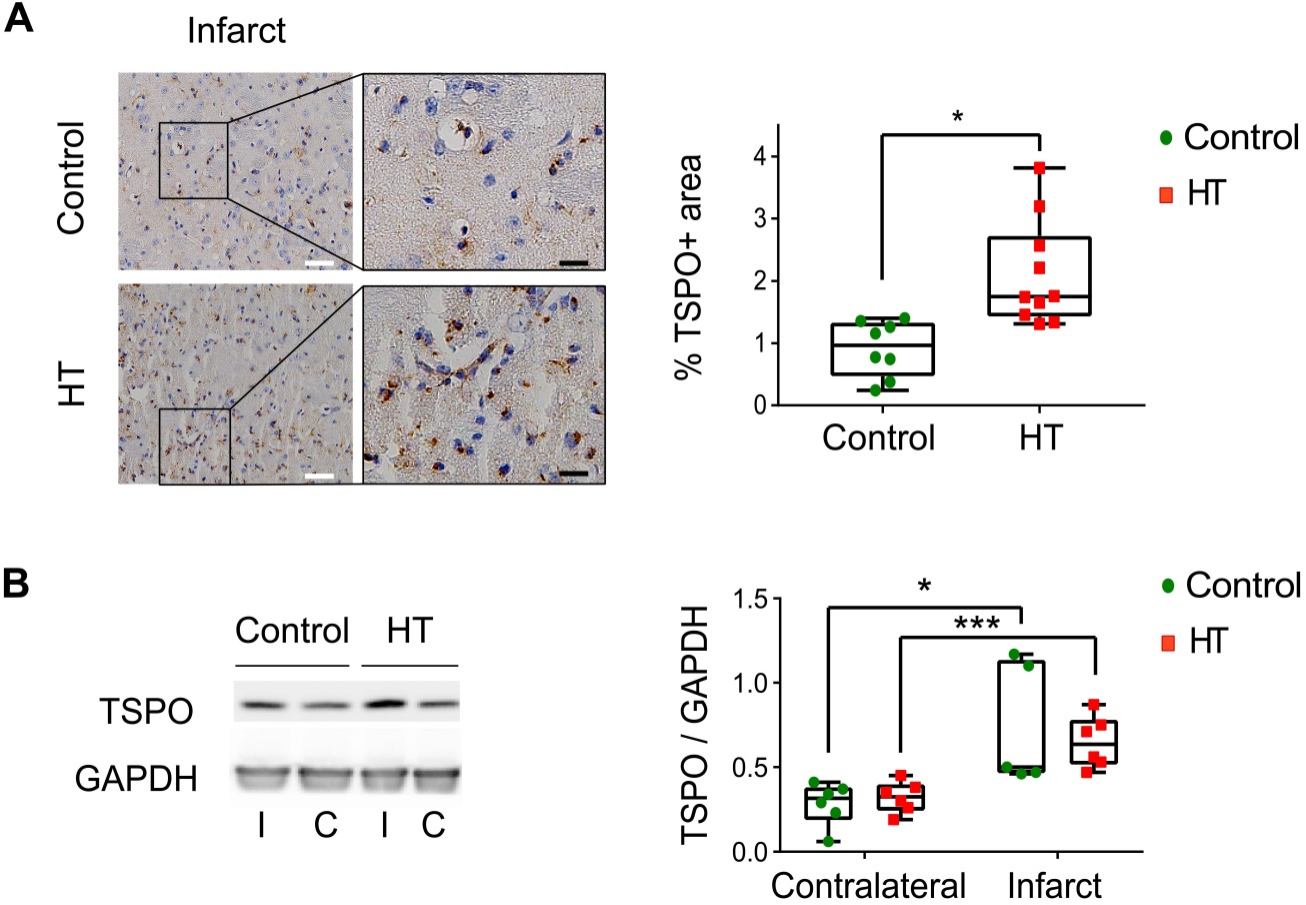
** A HT diet induces a slight increase in TSPO expression at day 35 post ischemia.** (A) Representative TSPO staining within the infarct and contralateral side obtained by immunohistochemistry at day 35 post ischemia from both control and HT-fed mice (white scale bar: 30 µm, black scale bar: 8 µm). For both experimental groups, immune cells and endothelial cells showed TSPO immunoreactivity. A HT diet slightly increased TSPO-related inflammatory level (n = 3/group, with 3 fields of view per animal). (B) Representative western blot for TSPO from brain protein extracts obtained at day 35 post ischemia (n = 6/group). A HT diet did not change TSPO protein levels in both the infarct (I) and contralateral (C) hemispheres at day 35 post ischemia. Values are normalized relative to GAPDH band. Statistical analysis was carried out with two-way ANOVA for multiple comparisons followed by Holm Sidak's post hoc test for multiple comparisons. Values represent mean ± sd. (*p < 0.05, ** p < 0.01, ***p < 0.005; * *vs.* contralateral, # *vs.* diet).

We cross-validated [^18^F]DPA-714 PET images with TSPO by immunohistochemistry (IHC) and western blot (Figure 6, Figure S6).

The stroke effect on TSPO expression levels was observed by IHC, indicated by significant higher percentage of stained area within the infarct compared to the contralateral side for both dietary groups (Figure 6A). Dietary effect was observed within the infarct, indicated by a slight increase in TSPO expression in HT-fed mice (2.11 ± 0.8%) compared to control mice (0.91 ± 0.42%, *p* = 0.011) (Sidak's post hoc test, *p* < 0.001) (Figure 6A) while no dietary effect was observed in the contralateral side (control: 0.40 ± 0.09%, HT: 0.44 ± 0.13%, *p* = 0.83).

On the other hand, no significant difference in TSPO expression at the protein level (western blot) was observed between the treatment groups, in both infarct (control: 0.74 ± 0.36, HT: 0.65 ± 0.15, Sidak's post hoc test, *p* = 0.89) and contralateral (control: 0.28 ± 0.13, HT: 0.32 ± 0.09, Sidak's post hoc test, *p* = 0.97) hemispheres (Figure 6B).

### Cellular localization of TSPO expression within the ischemic lesion at day 35 post ischemia

**Figure 7:**
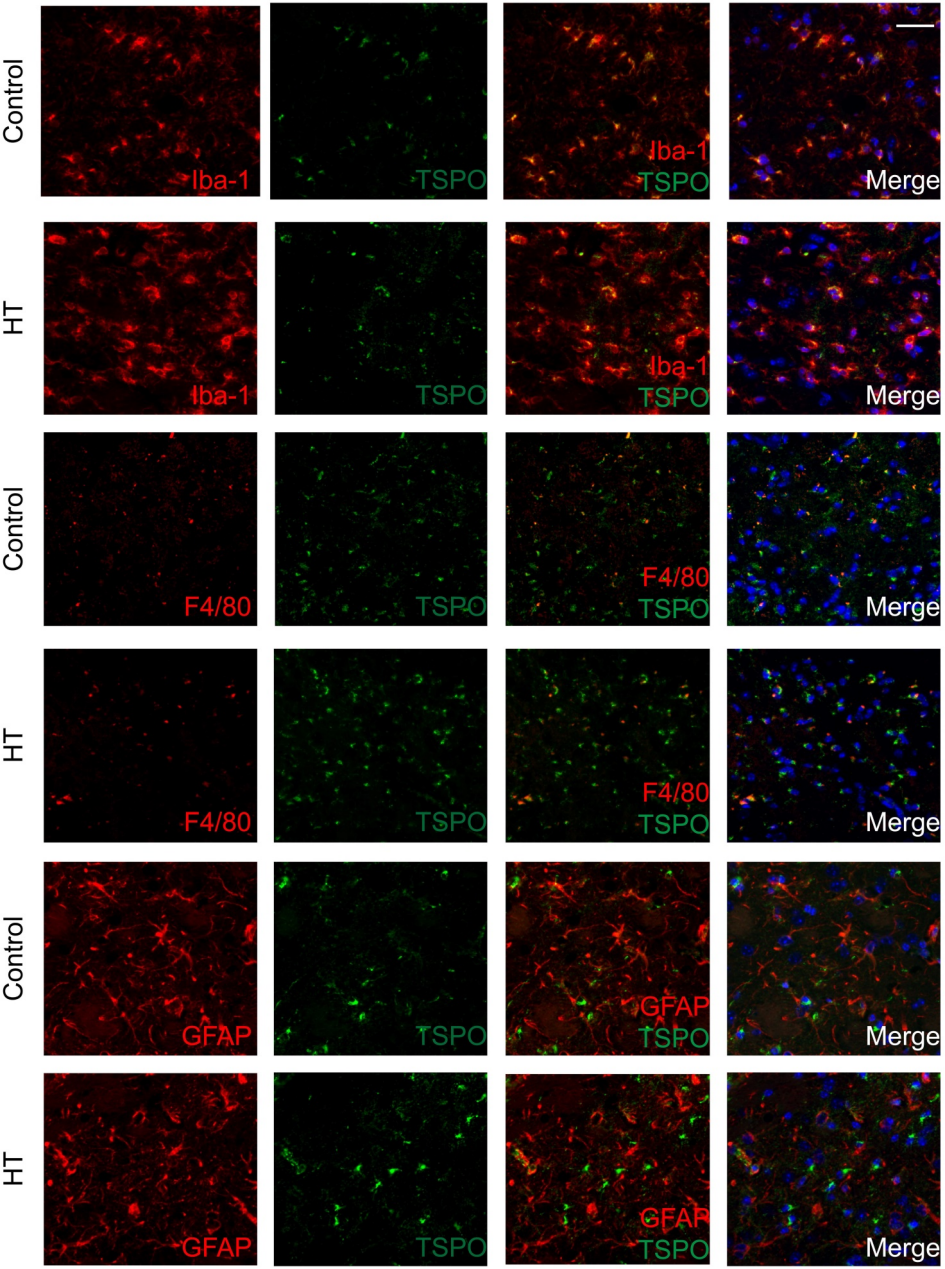
** TSPO is mainly expressed in microglia/macrophages within the infarct at day 35 post ischemia.** Co-localization of TSPO with immune cells markers such as Iba-1, GFAP and F4/80 at day 35 post ischemia highlighted strong microgliosis independent of TSPO expression. No or minor colocalization of GFAP or F4/80 positive cells with TSPO was found. Nuclei are counterstained with DAPI (blue). White scale bar: 40 µm.

The brains were double stained for TSPO and Iba-1 or GFAP or F4/80 at day 35 post ischemia to identify the cellular sources of TSPO. Co-localization of different cell markers indicated TSPO to be partly expressed by Iba-1^+^ and F4/80^+^ (microglia/macrophages) cells for both experimental groups. TSPO expression was not found in GFAP^+^ (astrocytes) cells in both dietary groups (Figure 7).

Quantification of the images is reported in Figure S7. For both Iba-1^+^ and F4/80^+^, treatment effect was observed on TSPO^-^ cells, indicated by the significant increase of Iba-1^+^TSPO^-^ and F4/80^+^TSPO^-^ (*p* < 0.005) with HT. Besides, HT significantly increased the number of Iba-1^+^CD163^+^ cells (*p* = 0.01).

### Hydroxytyrosol extensively increases the number of Iba-1 positive cells within the infarct at day 35 post ischemia

**Figure 8:**
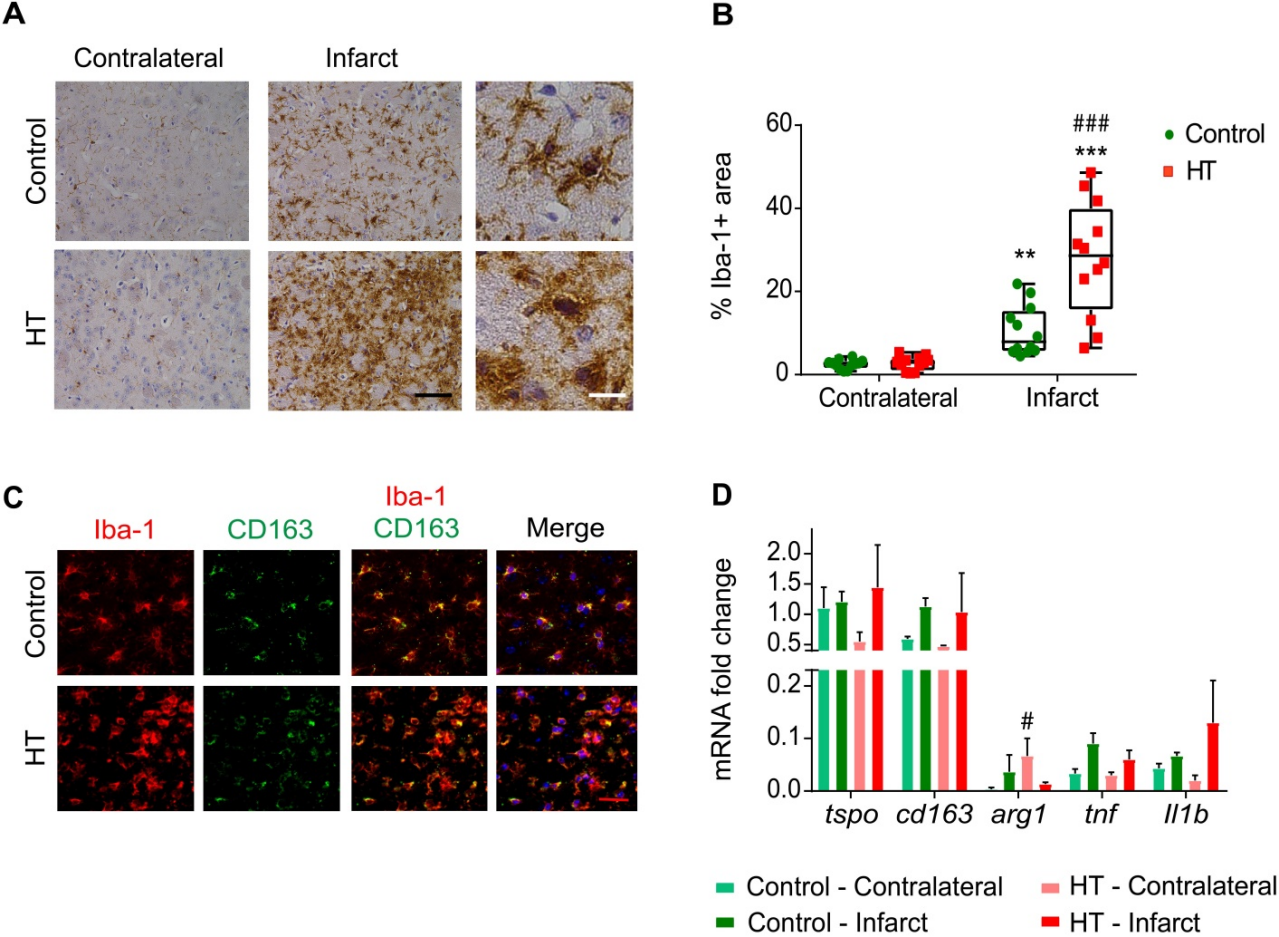
** HT increases Iba-1 positive cells number within the infarct at day 35 post ischemia.** (A) Representative immunohistochemistry for Iba-1 in brain of control (upper row) and HT-fed (lower row) mice within the infarct and at the contralateral side 35 days after tMCAo. Iba-1 positive cells within the ischemic lesion exhibited an amoeboid morphology in brain of HT-fed mice compared to control mice brain (ramified shape) (black scale bar: 50 µm, white bar: 9 µm). (B) Quantification of the percentage of Iba-1 stained area showed increased Iba-1 positive area within the infarct in HT-fed mice (red) compared to controls (control), indicative of HT's immunomodulatory effect on Iba-1 positive cells (n = 4/group, with 3 fields of view per region). (C) Most of the Iba-1 positive cells co-expressed CD163, an anti-inflammatory marker (red scale bar: 10 µm). (D) Gene expression confirmed immunofluorescence data and indicated a trend toward an increased expression of anti-inflammatory markers after HT treatment at day 35 post ischemia. Statistical analysis was carried out with two-way RM ANOVA followed by Holm Sidak's post hoc test for multiple comparisons (*p < 0.05, **p < 0.01, ***p < 0.005, * *vs.* contralateral, # *vs.* diet).

We found very prominent microglial activation within the infarct in HT-fed mice compared to control mice, as evidenced by histology (Figures 8A-B).

Stroke effect was observed in both groups (two-way RM ANOVA, *p* < 0.001): the percentage of Iba-1 positive area within the infarct was significantly increased compared to the contralateral striatum, for both control (*p* = 0.008) and HT-fed mice (*p* < 0.001) (Sidak's post hoc test) (Figure 8A).

A dietary effect was observed within the infarct (two-way RM ANOVA, *p* = 0.001): the percentage of Iba-1 positive area in HT-fed mice was higher compared to control mice (*p* < 0.001, Sidak's post hoc test). The same tendency was observed in brain tissue collected at day 7 post ischemia, where an increased percentage of Iba-1^+^ cells was found within the ischemia in HT-fed mice compared to control mice (Figure S8). Iba-1 positive cells within the ischemic lesion exhibited activated/reactive morphology with hypertrophic cell bodies and very short processes in brain of HT-fed mice in comparison to the more ramified morphology of Iba-1 positive cells in control mice brain (Figure 8A). No diet effect was observed in the contralateral side (*p* = 0.98, Sidak's post hoc test).

The correlation between TSPO and Iba-1 expression indicated a linear relationship between both markers (R^2^ = 0.80, Figure S9).

To investigate the anti-inflammatory potential of HT, we characterized the Iba-1^+^ cells population co-expressing CD163, a general anti-inflammatory marker (Figure 8C). We observed a significant treatment effect on the number of Iba-1^+^CD163^+^ cells (Figure S7B), indicating that HT may increase the number of anti-inflammatory microglia/macrophages at late time points.

Besides, we investigated gene expression of pro- and anti-inflammatory markers at day 35 post ischemia (Figure 8D). No significant treatment effect was observed on *tspo*, *cd163*, *tnf* and *Il-1β* gene expression in both infarct and contralateral hemisphere (*p* > 0.05). Significant increase of the anti-inflammatory marker arginase 1 (*arg1*) was observed in the contralateral hemisphere (Sidak's post hoc test, *p* = 0.043).

### Hydroxytyrosol improves strength recovery within first two weeks after stroke

Functional deficits were assessed with a set of behavioural and motor tests in all mice before and after ischemia following the timeline shown in Figure 1.

### Open field (Figure 9)

**Figure 9:**
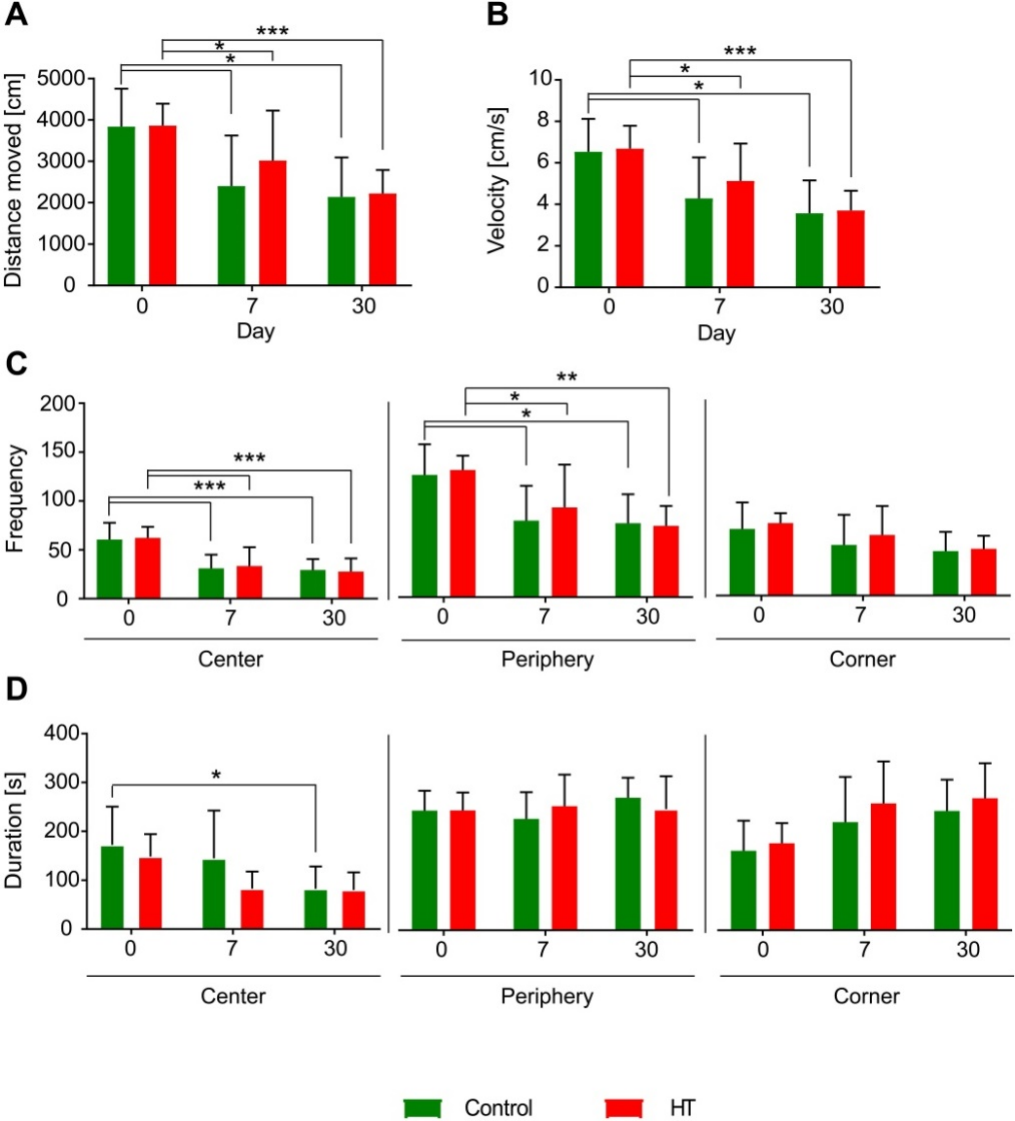
**Hydroxytyrosol does not improve locomotion.** Distance moved [cm], velocity [cm/s], frequency and duration [s] were assessed like index of locomotion and anxiety. Overall, there was no dietary effect observed on locomotor activity and anxiety level assessed by open field. The time spent in the central zone significantly decreased at day 30 post ischemia in control mice compared to baseline while no difference was observed in HT-fed mice. Values represent mean ± sd. Control: n = 8, HT: n = 8. Statistical analysis was carried out with two-way RM ANOVA followed by Holm Sidak's post hoc test for multiple comparisons (*p < 0.05, **p < 0.01, ***p < 0.005).

Exploratory behaviour, activity, and anxiety were assessed using the open field test (Figure 9). At days 7 and 30 post ischemia, all mice travelled a shorter distance (Figure 9.A) at a lower velocity (Figure 9B) compared to baseline. No difference was observed between days 7 and 30 in both groups. No dietary effects were observed on distance moved and velocity (two-way RM ANOVA, *p* > 0.05).

All mice visited less frequently the centre and the periphery of the arena at days 7 and 30 compared to baseline while no change was observed in the corners over time. No dietary effects were observed on the frequency of entrance in each zone (Figure 9C).

The time spent in the central zone significantly decreased at day 30 post ischemia in control mice compared to baseline (*p* = 0.048) while no difference was observed in HT-fed mice. No difference in time spent along the peripheral zones and the corners was observed over time for both dietary groups. No diet effect was observed in time spent in the different corners of the open field (Figure 9D).

### Grip strength test (Figure 10)

Forelimb (trapeze) and total-limb (grid) grip strength test revealed no significant difference in baseline level (day 0, D0) between groups, for both fore- and total limbs (*p* > 0.05).

**Figure 10:**
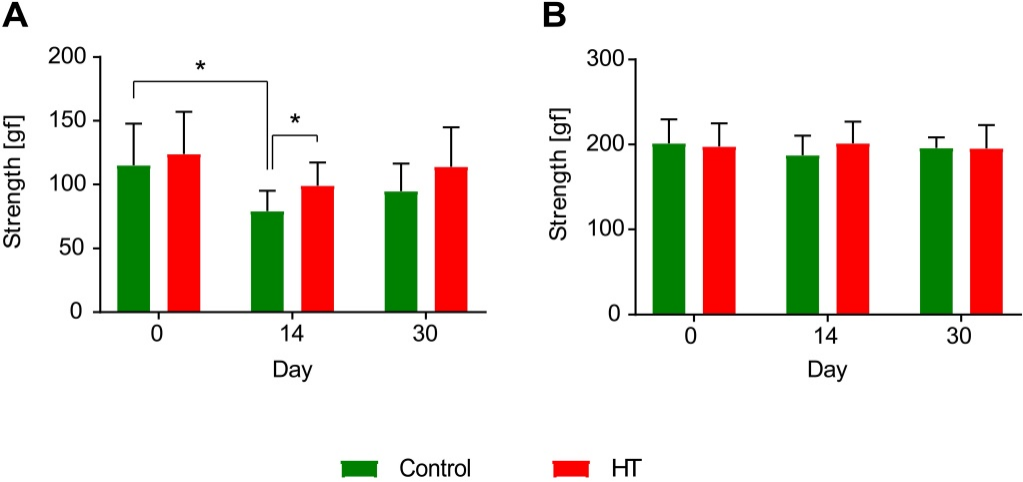
** Hydroxytyrosol improves strength recovery of the forelimbs after ischemia.** (A) Grip test revealed a HT diet improved forelimbs strength and thus recovery at day 14 post ischemia (*p* = 0.031). In control mice, the grip strength was decreased 14 days after stroke while no difference was observed in HT-fed mice (*p* = 0.031). (B) However, no difference was observed in total limbs strength over time. Values represent mean ± sd. Control: n = 8, HT: n = 8. Statistical analysis was carried out with two-way RM ANOVA followed by Holm Sidak's post hoc test for multiple comparisons (*p < 0.05).

Within group comparisons a significant reduction was observed in forepaws strength of control mice between baseline (D0: 115.0 ± 32.7 gf) and day 14 (D14: 80.5 ± 17.0 gf) post ischemia (two-way RM ANOVA, *p* = 0.031). Within the HT-fed mice group, no significant decrease in strength was observed between pre- and post-ischemia assessment (two-way RM ANOVA, *p* > 0.05). However, diet group comparisons indicated a significant improvement in grip strength in HT-fed mice when compared to control at day 14 post ischemia (two-way RM ANOVA, *p* = 0.031) (Figure 10A).

Within group comparisons total limbs strength baselines did not differ from the post-stroke follow up at both day 14 and 30 post ischemia (*p* > 0.05), for both control and HT-fed mice (Figure 10B).

## Discussion

The Mediterranean diet is recommended as a healthy dietary pattern to prevent cardiovascular diseases and neurodegenerative processes 22. Hydroxytyrosol, the main phenolic compound of the EVOOO, is well-known for its antioxidant and anti-inflammatory properties, protecting vascular health 47. In this context, HT dietary intervention seems a promising multifaceted approach to prevent but also to slow down the progression of cardio- and cerebrovascular diseases. In this study, we investigated the anti-inflammatory, cerebrovascular and functional outcomes after dietary treatment with HT in the post ischemic phase. HT strongly increased numbers of Iba-1^+^ microglia/macrophages population also expressing CD163 (anti-inflammatory marker) within the infarct region, inducing a slight increase in TSPO expression not detected by [^18^F]DPA-714 PET.

Interestingly, HT improved strength recovery within the first two weeks while no treatment effect was observed on the selected *in vivo* imaging parameters. Overall, we showed that a prolonged dietary intervention with HT modulated the glial response and enhanced muscle recovery during the post ischemic phase.

### Edema

Anti-inflammatory olive oil phenols have been considered as neuroprotective nutraceuticals usable in ischemic brain injury prevention. Mohagheghi et al. (2010) investigated the impact of dietary VOO on brain infarct and showed that 1-month pretreatment reduced infarct volume in rats 48. The same protective effect was observed with olive leaf extract 49 containing HT.

Here, T_2_w-MR imaging was used to delineate the infarct area following the method previously reported 43,50. No treatment effect was observed on the T_2_w-MR edema volume. While VOO and hydroxytyrosol administrations prior ischemia are able to decrease infarct volume, our data suggested no therapeutic effect of HT within the first week on infarct size.

Similarly, cytotoxic edema was identified by enhanced hyperintense signals on DW images. Our results also indicated no treatment effect on the mean ADC value over time. While pretreatment with HT-containing virgin olive oil strongly reduced brain free water content after MCAo in rats 51, HT as a therapy does not show any protective effect on edema and/or water compartmentalization after stroke.

### Vascular parameters

Vascular endothelial cells are central players in the inflammatory cascade. Under physiological conditions, they regulate blood flow and control vessel permeability. Following stroke, endothelial cells are activated by a plethora of signalling molecules, leading to a decreased CBF, increased vessel-wall leakage and subsequent infiltration of PNS immune cells at the site of inflammation. HT, as an antioxidant, has been reported beneficial against vascular diseases by improving endothelial functions 52. In *in vitro* models, HT suppressed inflammatory angiogenesis by inhibiting endothelial cell proliferation 27 and promoting apoptosis 53,54.

In our study, we did not detect any treatment effect on CBF. In a recent paper, Calahorra et al (2019) indicated that HT-fed mice had increased CBF in the right hippocampus and left cortex at days 7 and 35 post ischemia compared to control mice. However, the authors did not measure CBF within the infarct region.

Additionally, we used nitric oxide (NO) as a peripheral marker to assess vascular tissue at day 35 post ischemia. Lausada et al. (2015) showed that EVOO consumption prior and after experimental stroke was protective against iNOS activation in the penumbral region within the first days post ischemia (73). In our study, we did not observe any treatment effect on NO levels, as previously reported by Calahorra et al. (2019) 34. The peak of iNOS expression was observed within two weeks post ischemia but resolved after one month post ischemia (71,72), which may explain why we did not observe any effect at day 35 post ischemia.

### Anti-inflammatory properties

Stroke causes a strong inflammatory reaction within infarct and peri-infarct areas involving heterogeneous immune cell populations 55,56. Observations indicated that microglia/macrophages and astrocytes actively modulate the inflammatory cascade at different stages of the disease 56,57. They are initial responders to injury and contribute to cognitive deficit/recovery at later stages, depending on their activation state, ranging from a more pro-inflammatory to an anti-inflammatory state, modulating both neuronal cell death and tissue restoration, respectively 58,59.

Translocator protein (TSPO) PET imaging provides a non-invasive method to repeatedly access neuroinflammation/glial activity through the disease time course 55,60-62. TSPO is expressed at low level under normal physiological conditions while its expression increases in activated glial cells in response to brain injury 63. In a rat stroke model, [^18^F]DPA-714 (TSPO) signal within the infarcted area peaked around day 10-14 post ischemia, a signal mostly induced by microglia/macrophages 64 while a greater astrocytic contribution to the signal was observed at latter time points. Therefore, TSPO expression by microglia/infiltrating macrophages and astrocytes seems to be spatiotemporally dependent 62,64-66. However, its differential expression pattern and precise function in inflammatory conditions remain unclear.

The impact of HT on immune cells is still sparsely explored 34. Here, we showed that HT drastically increased the number of Iba-1^+^ cells (microglia/macrophages) within the infarcted brain area at day 35 post ischemia without any effect on the contralateral hemisphere. No significant difference was observed in F4/80 expression, a marker mostly upregulated in macrophages and at a low level in resting microglia, indicating that HT might preferentially stimulate the proliferation of microglial cells. However, further studies are needed to further characterize HT induced changes in the glial compartment at earlier time points. As a first indication, increased Iba-1 and CD68 immunoreactivity was also observed at day 7 post ischemia.

The accumulation of those numerous cells could not be detected by using [^18^F]DPA-714 (TSPO) PET imaging while a positive correlation was observed between the amount of Iba-1^+^ area and radiotracer uptake. Immunoreactivity is the gold standard to validate *in vivo* PET imaging due to its higher sensitivity. Our TSPO IHC data indicated a two-fold increase in TSPO expression (going from 1% to 2% TSPO^+^ area) in HT-fed mice while no significant changes were observed in TSPO-PET and WB. The detection of these changes presumably lies beyond the sensitivity of the PET technique. Additionally, small changes could also be hidden by partial volume effects (due to high uptake observed in surrounding glands and fatty tissues), the low number of animals and/or the inherent differences in quantification methods (2D *vs.* 3D). Further studies are needed to characterize HT induced changes in the glial compartment on a tissue level at earlier time points after stroke.

In a previous study, we investigated the neurorestorative efficacy of a dietary approach on TSPO expression as well 46. Results indicated that *in vivo* DPA-714 PET was able to detect therapy effect on TSPO expression at late time points. However, the diet used in the latter study was a multicomponent diet that might induce a stronger therapeutic effect compared to our single component diet. Thus, we hypothesise that the dynamic range of changes on TSPO expression induced by a HT diet might be hidden by the stroke effect and PET sensitivity. HT slightly stimulates TSPO overexpression on a tissue level.

To our knowledge, it remains unclear if a larger Iba-1^+^ cell population is more beneficial than resting microglial cells at late stages, while “active-shaped” microglia indicated homeostatic imbalance. Here, our results indicated that there was no benefit from late “activated” microglial on stroke outcomes. However, Lartey et al. (2014) demonstrated a positive correlation between three microglial related proteins (TSPO, CD11b and CD68) and motor function (rotarod) over the first 22 days following stroke in mice 67, indicating that better motor outcome is associated to increased microglial activity and/or number.

We further characterized the Iba-1^+^ cell populations and the inflammatory environment by assessing the expression level of common markers. CD163 gene expression was similar in both groups as its fluorescence intensity, suggesting that the apparent increase in CD163 in the HT group was related to the overall increase in Iba-1^+^ cells. Moreover, the increased number of Iba-1^+^(CD163^+^) cells in the HT-fed group may partly explain the increase in Arg1 and Il-1β gene expression. Overall, our results supported the potential anti-inflammatory effect of HT during the post ischemic phase, indicated by higher expression of anti-inflammatory markers, while no clear-cut effect was observed. Future investigations should focus on a more extensive evaluation of the anti- and pro-inflammatory microglial polarization markers using a dedicated study design and appropriate techniques (scRNA and spatial transcriptomics) to conclude on the potential immunomodulatory effect of HT.

Cellular and scarce preclinical studies further support the anti-inflammatory properties of HT 22,30,68. Among others, the mechanisms by which HT is able to prevent and counteract inflammation include reducing oxidative stress and free radical production 30. Pretreatment with HT was shown to inhibit cyclooxygenase-2 (COX-2) and iNOS genes expression in LPS challenged J774 murine macrophages 30 while no effect was observed when HT was added 12 hours after LPS. Besides, HT pre-cultured RAW264.7 murine macrophages cells also showed that HT decreased NO, prostaglandin E2 (PGE2) production and secretion of pro-inflammatory cytokines such as IL-1β, IL-6 and TNF-α 23 in a concentration-dependent manner. In a mouse model of systemic inflammation, pretreatment with HT also reduced COX-2 and TNF-α 29.

Altogether, these studies support the preventive rather than the curative effects of HT on the inflammatory cascade. Accordingly, the famous PREDIMED clinical study reported the preventive effect of the Mediterranean diet against stroke and cardiovascular events 26 while its therapeutic effect has been scarcely investigated. We do believe that HT, as a single component diet, has a weaker effect than a multicomponent diet containing other Mediterranean components. As stated before, in our previous work, we investigated the therapy effect of a multicomponent diet on stroke outcomes and reported significant effects on brain inflammation 46. Therefore, we do support the use of the Mediterranean diet as a therapeutic approach since HT, as a single component, already showed interesting modulatory effects on stroke outcomes.

### Antioxidant properties

The generation and release of oxygen free radicals (ROS) increase in acute disease is leading to high oxidative stress, associated with increased cellular death, blood-brain barrier disruption and inflammation 69.

One of the main beneficial health effects of HT was so far related to its ROS-scavenging properties and its ability to activate endogenous antioxidant systems. The potential antioxidant effect assessed by the level of the lipid peroxidation biomarker thiobarbituric acid reactive substances (TBARS) in serum did not indicate a significant difference between treatment groups at day 35 post ischemia. We suppose that release of ROS/RNS is an early process that is already resolved at the late stages of the disease 34,70.

## Limitations of the study

First, the therapeutic efficiency is dependent on the dietary intake. Within the first week, the animals reduced their food intake and lost weight. Therefore, the potential acute therapeutic effect of HT on oxidative stress and inflammation could be underestimated within the first week.

The sample size was calculated based on a previous study where we investigated the therapeutic effect of a dietary approach on [^18^F]DPA-714 tracer uptake in ischemic mice. Moreover, the same groups were used to perform MR imaging and behavioural tests. Due to the large variability between animals in behaviour, the statistical power of the different tested parameters is below 0.80. Post-analysis of the data indicated that statistical power for the velocity assessed by the pole test and strength were of 0.84 and 0.71, respectively. However, the statistical power was on the low side for rotarod and open field, with 0.36 and 0.63 respectively. Therefore, the protective potential of HT on cognition and motor functions might be under- and/or overestimated. In our study, we showed that a prolonged HT treatment in the post ischemic phase improved forepaw strength recovery (power: 0.71). This finding was supported by previous stroke studies 55, 72, that observed improved motor functions after a single injection of HT in a 120 min tMCAo rodent model 55 and improved forelimbs strength in the acute phase of the disease 55. Overall, we concluded that HT could improve motor functions.

## Conclusion

The understanding of how nutrition and in particular components of the Mediterranean diet may help treatment after brain injury has grown in recent years. In this context, we performed a longitudinal *in vivo* imaging study of the neuroinflammation, cerebrovascular and functional outcomes after a prolonged HT-enriched diet in an experimental stroke model. Data presented in this work indicated that HT has immunomodulatory effects by increasing the number of Iba-1^+^ microglia/macrophages in the post ischemic tissue. Analysis of inflammation-related markers expression supported that HT may favour an anti-inflammatory state. Further studies should focus on in-depth characterization of the pro- and anti-inflammatory markers expression using dedicated techniques (scRNA and spatial transcriptomics).

Overall, molecular changes induced by HT, as a single component of the Mediterranean diet, were not detected by *in vivo* imaging while we did observe a significant positive effect on muscle strength recovery. Therefore, we do support the use of the Mediterranean diet as a therapeutic approach since HT, as a single component, already showed interesting molecular modulatory effects on stroke outcomes.

## Supplementary Material

Supplementary figures and tables.

## Abbreviations

ANOVA: analysis of variance
CBF: cerebral blood flow
cm: centimetre
DPA: N,N-diethyl-2-[4-(2-fluoroethoxy)phenyl]-5,7-dimethylpyrazolo[1,5-a]pyrimidine-3-acetamide
DTI: diffusion tensor imaging
DWI: diffusion weighted imaging
EPI: echo-planar imaging
FOV: field of view
GAPDH: glyceraldehyde 3-phosphate dehydrogenase
GFAP: glial fibrillary acidic protein
HT: hydroxytyrosol
IBA-1: ionizing calcium-binding adaptor
ID: injected dose
ml: millilitre
MRI: magnetic resonance imaging
OF: open field
PBS: phosphate-buffered saline
PET: positron emission tomography
PFA: paraformaldehyde
PWI: perfusion-weighted imaging
RM: repeated measures
RT: room temperature
s: second
sd: standard deviation
tMCAo: transient middle cerebral artery occlusion
TSPO: translocator protein
T_2_w: T_2_-weighted
^18^F: fluorine-18
