# Supplementary material for: Impact of hydroxytyrosol on stroke: tracking therapy response on neuroinflammation and cerebrovascular parameters using PET-MR imaging and on functional outcomes
Source: Theranostics. 2021 Feb 15;11(9):4030–49. doi: 10.7150/thno.48110 (PMC7977466; doi:10.7150/thno.48110)
Supplement: Supplementary file 1 — Supplementary figures and tables. [file thnov11p4030s1.pdf]

Supplementary Material and Methods

Magnetic resonance imaging

Magnetic resonance imaging (MRI) scans, including T_2-weighted (T_2w), diffusion weighted imaging (DWI) and perfusion weighted imaging (PWI), were conducted at day 1, 3, 7, 14, 21 and 30 after tMCAo.

First, animals were placed in an oxygen-enriched box supplied with 5% isoflurane (Forene, Abbott, Wiesbaden, Germany). Then, mice were positioned on a heated MRI cradle, fixed by bite and ear bars and continuously supplied with 2 % isoflurane in air until the end of the experiment. Respiration and body temperature (37.0 ± 0.5°C) were constantly monitored. A small sheet prepared from 1% v/v agar-water solution was directly placed on the animal head to reduce susceptibility artefacts and covered with parafilm (Merck KGaA, Darmstadt, Germany). A 2 mm-surface coil (Bruker, Ettlingen, Germany) was positioned above the parafilm and fixed with tape. The cradle was manually positioned in the center of the 9.4 T small animal MRI scanner (Biospec 94/20, Bruker Biospin GmbH, Ettlingen, Germany). All images were processed and generated using Paravision 5.1 (Bruker Biospin MRI, Ettlingen, Germany).

T_2-weighted (T_2w) images were acquired with a fast spin-echo sequence (RARE) (Repetition Time (TR) = 2000 ms, Echo Time (TE) = 75 ms, Field Of View (FOV) = 2 cm x 2 cm, slice thickness = 0.5 mm, interslice distance = 0 cm, number of slices = 20, matrix = 192 x 192, number of averages = 8) to assess infarct size. Experimenter chose a single section, in the middle of the infarcted tissue, as reference for all the other MR images. The same slice location was prescribed for all the subsequent MRI sessions.

Diffusion-weighted echo-planar imaging (DTI EPI) (TR = 2500 ms, TE =31.30 ms; b-values = 100, 200, 400, 600, 800, 1000, 1200, 1600, 2400 [s/mm^2], FOV = 2 cm x 2 cm, slice thickness = 1.17 mm, matrix = 128 x 128, number of averages = 8) was performed to assess tissue architecture. Quantitative apparent diffusion coefficient (ADC) maps were calculated and generated.

Cerebral blood flow (CBF) was measured using the arterial spin labeling (ASL) method. The flow sensitive alternating inversion recovery rapid acquisition with relaxation enhancement (FAIR-RARE) was used to implement ASL with the following parameters: TR = 10000 ms, TE = 5.01 ms, FOV = 1.4 cm × 1.4 cm, slice thickness = 1 mm, number of

slices = 1, matrix = 64 × 64. The perfusion map was calculated using the ASL_Perfusion_Processing macro in ParaVision 5.1 (Bruker Biospin MRI, Ettlingen, Germany).

Immunohistochemistry/Immunofluorescence

5 µm sections from mouse brains fixed with 4 % paraformaldehyde and embedded in paraffin were used for immunohistochemical/immunofluorescent analysis.

After deparaffinization, heat-mediated antigen retrieval was performed by soaking the slices into a citrate buffer solution (pH = 6.0), for 2x 6 min at 360°C.

Immunohistochemistry was performed as follows: slices were soaked into a hydrogen peroxide (Wako, Neuss Germany) solution for 10 minutes at room temperature (RT) for blocking endogenous peroxidase and then treated with blocking solution for 1 hour at RT (5 % foetal bovine serum, 0.5 % Triton-X in PBS). Slices were incubated with primary antibody diluted in blocking solution overnight at 4°C, then soaked with the respective biotinylated secondary antibody and combined with streptadivin-HRP (in PBS, 30 minutes, RT). Sections were incubated with the DAB (3,3′-Diaminobenzidine) solution (in PBS) for few minutes at RT and washed. Slices were counterstained with hematoxylin and mounted using Entellan (Merck, Darmstadt, Germany).

Double-label immunofluorescence staining was performed as follows: brain sections were blocked with 5 % goat serum and incubated with a cocktail of primary antibodies overnight at 4°C. Sections were incubated with the associated conjugated secondary antibodies for 45 minutes at room temperature. Slides were mounted in DAPI containing mounting medium (Vectashield, H-1500, Vector Laboratories, Burlingame, CA, USA).

Primary antibodies and dilutions used were: anti-PBR antibody (anti-PBR, TSPO) (1:250, ab109497, Abcam Inc.), anti-ionized calcium biding adapter molecule 1 (anti-Iba1) (1:500, 019-19741, Wako Chemicals USA, Inc. Richmond, VA, USA), anti-glial fibrillary acidic protein (anti-GFAP) (1:500, ab4675, Abcam Inc.) and anti-F4/80 (1:200, ab6640, Abcam Inc).

Secondary antibodies and dilutions (diluted 1/800 in blocking solution) used were: goat anti-rabbit Alexa Fluor 555 conjugated (A-21432, Life Technologies, Carlsbad, CA, USA), goat anti-rabbit Alexa Fluor 488 conjugated (A-21206, Life Technologies, Carlsbad, CA, USA), goat anti-chicken Alexa Fluor 488 conjugated (A-11039, Life Technologies, Carlsbad, CA, USA), goat anti-rat Alexa Fluor 488 conjugated (112-545-167, Jackson Dianova, Hamburg, Germany).

Sections were viewed with a combined fluorescent-light microscope (Nikon Eclipse NI-E, Nikon, Tokyo, Japan).

Table S1. Study design and animal number. All *in vivo* assessment including PET and MRI were performed on the same stroke mice (with n = 8/ group, Group A & C). At day 35 post ischemia, those animals were sacrificed and n = 6 mice were used for histological characterization of the inflammatory response and n = 2 were used for semi-quantitative western blot. Extra groups (B&D) were added to increase the animal number for western blot. Groups B&D were not used for *in vivo* imaging.

Besides, n = 4 mice per group were added to the overall study to characterize the inflammatory environment by real time qPCR (RTqPCR). Those samples were provided by A. J. Kiliaan's group (reported in Calahorra et al. (2019)). Results are shown in the Supplementary figure S7.

**Diet**	**Group**	**PET/MRI Behaviour**	**Histology** (Day 35)	**Western Blot** (Day 35)	**RT-qPCR** (Day 35)
Control (n = 11)	A	8	6	2	
	B			3	
	C				4
HT (n = 11)	C	8	6	2	
	D			3	
	C				4
		**Total**	n = 6 per group	n = 5 per group	n = 4 Per group

Supplementary figures

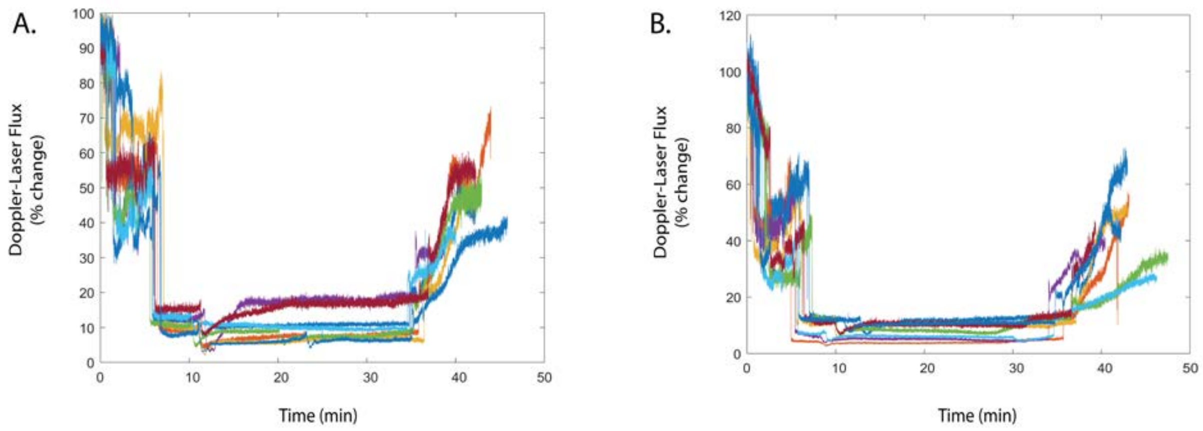

Figure S1. Percentage of Doppler-laser flux changes during a 30 minutes transient middle cerebral artery occlusion in (A) control and (B) HT fed mice groups.

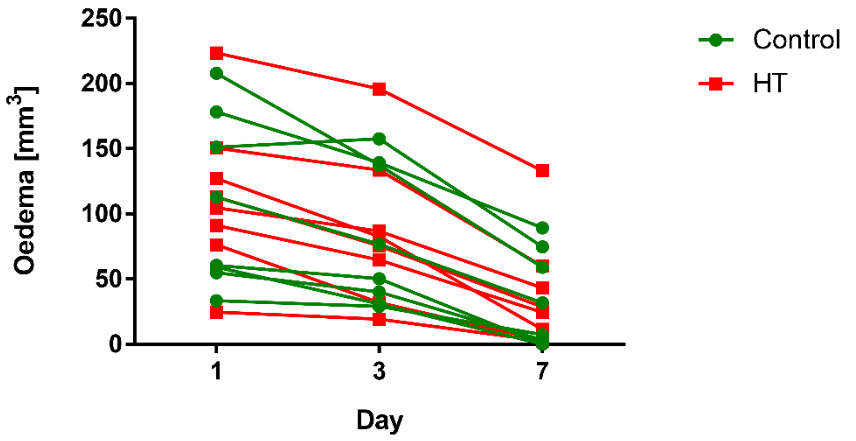

Figure S2. Individual edema/lesion recovery assessed by T2-weigthed magnetic resonance imaging. Edema volume was assessed in a total of n = 16 mice at day 1, 3 and 7 post ischemia by T2-weigthed MR imaging. Both control (green) and HT fed (red) mice similarly recovered, indicating that a HT diet does not affect the edema recovery rate.

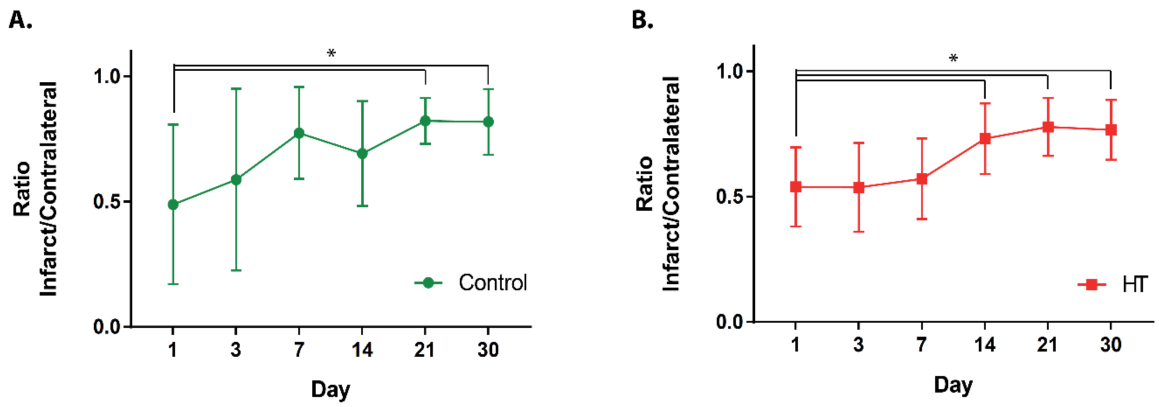

Figure S3. Infarct-to-contralateral CBF ratios in control and HT fed mice after ischemia. Infarct-to-contralateral CBF ratios dataset passed the Shapiro-Wilk (p = 0.35) and Brown-Forsythe (p = 0.93) tests. Two-way ANOVA indicated main effect of time (p < 0.001) but not of the treatment (p = 0.43) or interaction (p = 0.69). **(A)** In control mice, CBF ratios were significantly higher at day 21 (0.82 ± 0.10, Sidak's post hoc test, p = 0.04) and 30 (0.82 ± 0.13, Sidak's post hoc test, p = 0.021) compared to day 1 (0.49 ± 0.32) post ischemia. **(B)** Significant higher CBF ratio values were already seen at day 14 (0.73 ± 0.14) compared to day 1 (0.53 ± 0.16, Sidak's post hoc test, p = 0.023) in HT fed mice, while treatment effect was observed at any time point.

Values represent mean ± sd. Statistical analysis was carried out with two-way RM ANOVA followed by Holm Sidak's post hoc test for multiple comparisons. Control: n = 8, HT: n = 8. (*p < 0.05, * vs day 1)

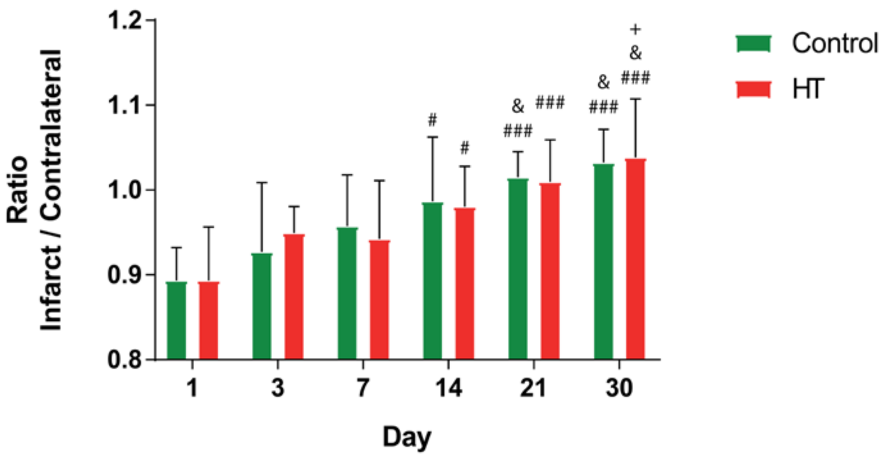

Figure S4. Infarct-to-contralateral ADC ratios in control and HT fed mice after ischemia. Infarct-to-contralateral ADC ratios dataset passed the Shapiro-Wilk ($p = 0.20$) and Brown-Forsythe ($p = 0.17$) tests. Two-way RM ANOVA indicated main effect of time ($p < 0.001$) but not of the treatment ($p = 0.81$) or interaction ($p = 0.82$). Infarct-to-contralateral ratio on day 1 post ischemia was significantly increased compared to day 14 ($p < 0.001$), 21 ($p < 0.001$) and 30 ($p < 0.001$) (Sidak's post hoc test). Besides, ADC ratios on day 3 and 7 were significantly increased compared to day 30 and/or day 14 post ischemia, indicating that ADC ratios recovered within days 7 and 14 post ischemia.

Values represent mean ± sd. Statistical analysis was carried out with two-way RM ANOVA followed by Holm Sidak's post hoc test for multiple comparisons. Control: n = 8, HT: n = 8. (* $p < 0.05$, ** $p < 0.01$, *** $p < 0.005$, * *vs.* treatment, # *vs.* day 1, & *vs.* day 3 and + *vs.* day 7).

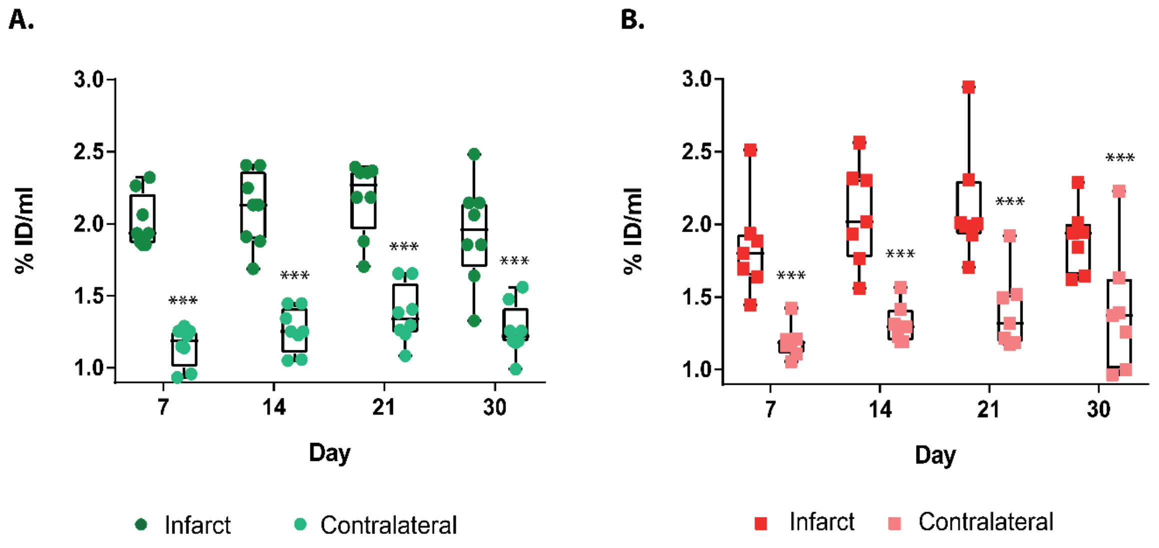

Figure S5. [^{18}F]DPA-714 uptake within the T2w-MR infarct and contralateral striatum after a tMCAo (30 minutes). *In vivo* [^{18}F]DPA-714 PET imaging revealed TSPO levels to be significantly increased within the infarct compared to the contralateral striatum at each time point. In both **(A)** control and **(B)** HT-fed mice, the two-way RM ANOVA revealed a significant effect of the region ($p < 0.001$) but not of time (control: $p = 0.36$; HT: $p = 0.15$) or interaction region * time (control: $p = 0.59$; HT: $p = 0.71$).

Values are expressed in percentage of injected dose by millimetre (%ID/ml). Data was analysed by two-way RM ANOVA followed by Sidak's post hoc test for multiple comparisons (*** $p < 0.005$ vs infarct).

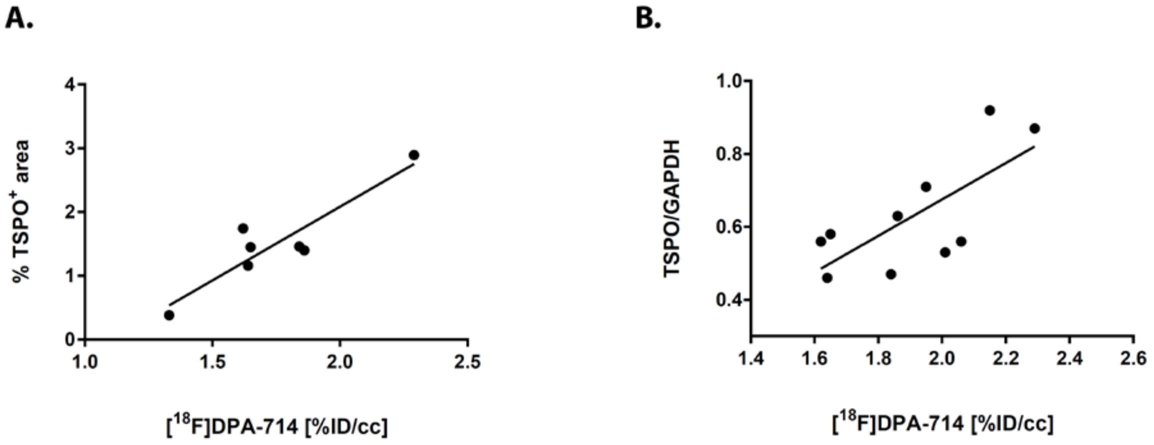

Figure S6. Cross-validation of [18F]DPA-714 PET images with TSPO expression measured by (A) immunoreactivity and (B) western blot. (A) Increased percentage of TSPO positive area and (B) normalized TSPO protein expression linearly correlated with [18F]DPA-714 uptake within the infarct on the last day of experiment (A : R^2 = 0.84, B : R^2 = 0.65).

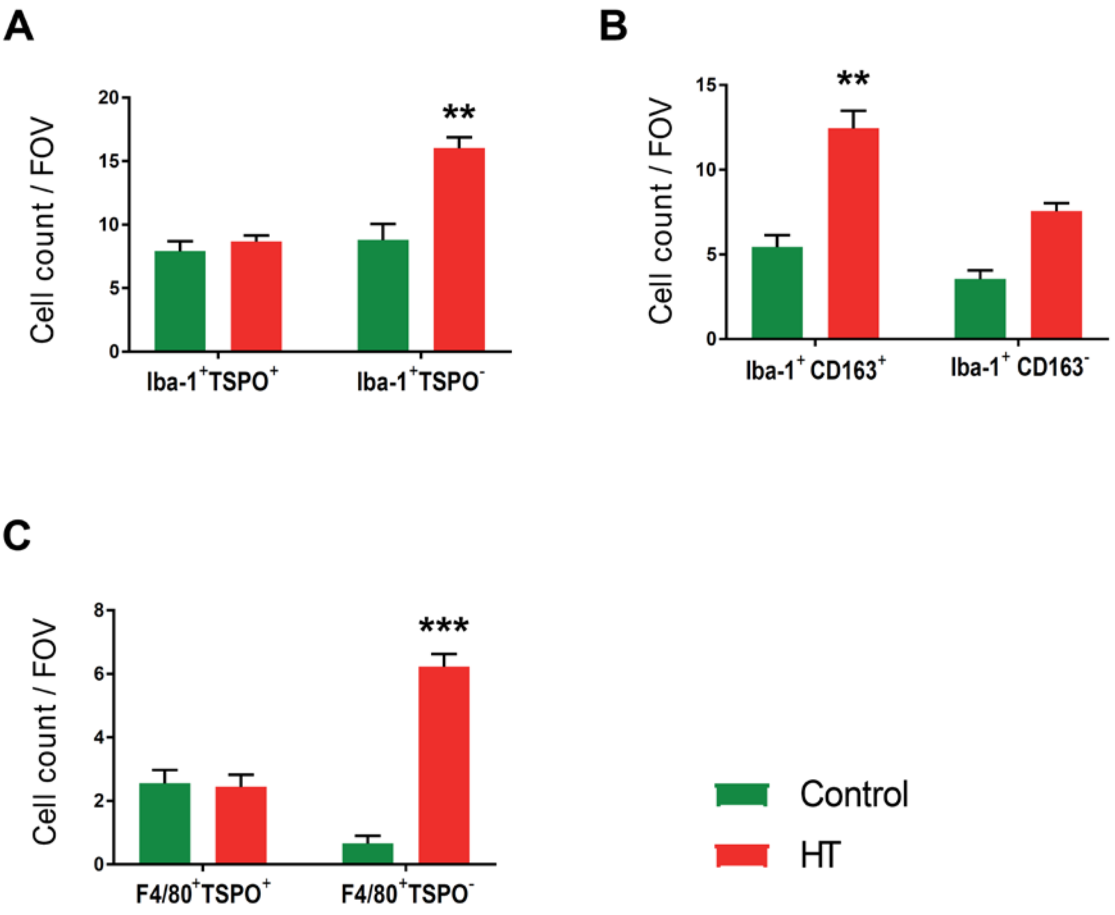

Figure S7. Quantification of immunofluorescence. Quantification of (A) Iba-1$^+$TSPO$^+$, (B) Iba-1$^+$CD163$^+$ and (C) Iba-1$^+$ F4/80$^+$ cells within the infarct and negative control cells at day 35 post ischemia. Number of Iba-1$^+$TSPO$^+$ and F4/80$^+$TSPO$^+$ cells did not differ between groups while TSPO$^-$ cells were significantly increase with HT. On the other hand, the number of Iba-1$^+$CD163$^+$ was significantly increased with HT while no difference was observed on Iba-1$^+$CD163$^-$ cells.

Values represent mean ± SEM (* $p < 0.05$, ** $p < 0.001$, *** $p < 0.005$, * *vs.* treatment). Statistical analysis was carried out with RM ANOVA followed by Holm Sidak's post hoc test for multiple comparisons.

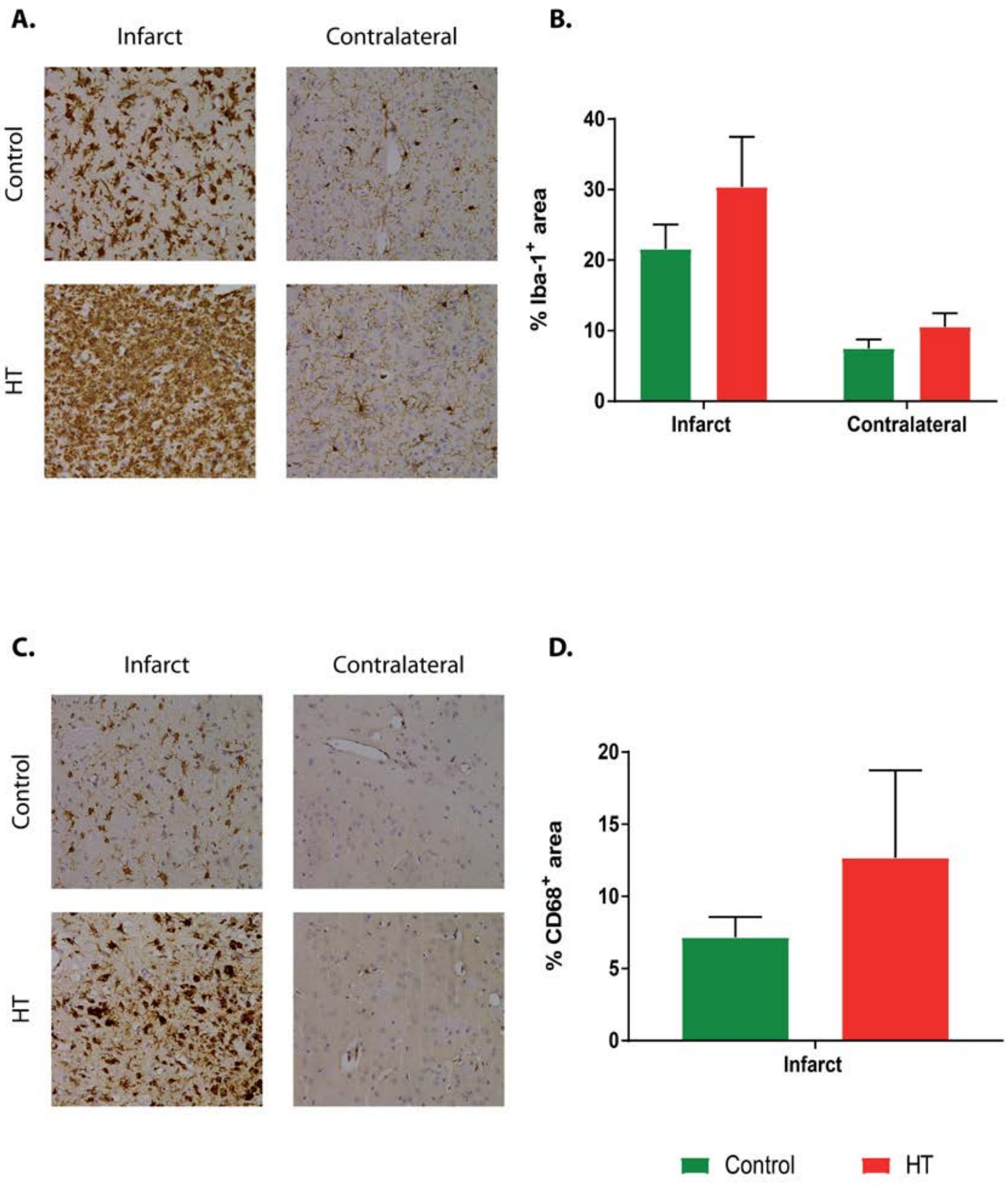

Figure S8. Iba-1 and CD68 expression at day 7 post ischemia detected by immunohistochemistry. (A) Representative immunohistochemistry for Iba-1 and (D) CD68 (activation marker) in brain of control and HT-fed mice within the infarct and in the contralateral side 7 days after tMCAo. Quantification of the percentage of (B) Iba-1 and (D) CD68 positive areas in control (green) and HT-fed mice (red).

No statistical analysis was carried out because due to the limited availability of tissue (control: n = 4, HT: n = 2).

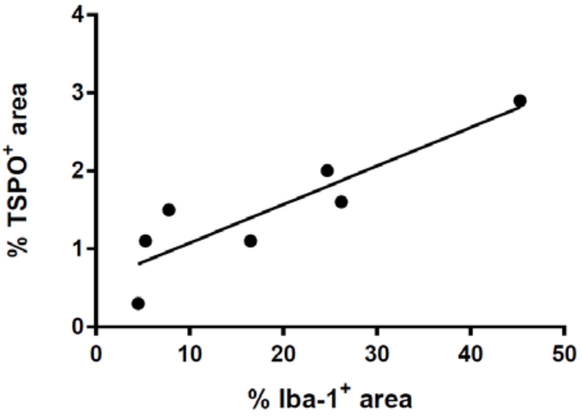

Figure S9. Correlation between the percentage of TSPO+ and Iba-1+ area measured by immunoreactivity. Increased percentage (%) of Iba-1+ area linearly correlate with increased % of TSPO+ area within the infarct at day 35 post ischemia (R^2 = 0.80).

Blood plasma analysis

We assessed levels of nitric oxide (NO, [μM]) and thiobarbituric acid reactive substances (TBARS) in serum samples at day 35. No significant difference was detected between control and HT-fed mice for NO (t-test, p = 0.057) and TBARS (t-test, p = 0.23).

A. **B.**

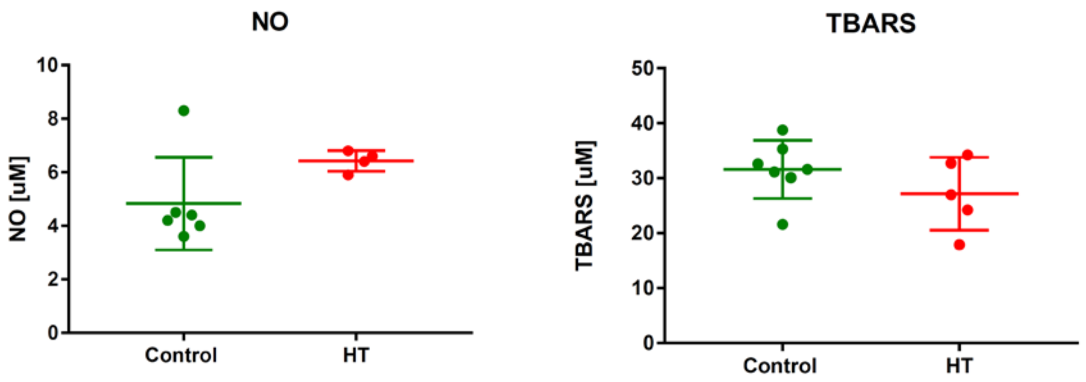

Figure S10. Nitric oxide (NO) and thiobarbituric acid reactive substances (TBARS) in serum samples at day 35 post ischemia. (A) NO levels are similar in control and HT-fed mice at day 35 post ischemia. (B) Levels of lipid peroxidation in control and HT indicate no dietary effect on peripheral ROS levels.

Behavioural test - Rotarod

Rotarod test was performed to assess dietary effects of HT on coordination and balance.

Baseline levels of run distance (day 0) did not differ between groups (control: 11. 3 ± 6.2 cm, HT: 10.2 ± 4.5 cm). Baselines did not significantly differ from the post-stroke evaluation at day 14 and 30 post ischemia (two-way RM ANOVA, time, p = 0.65). No dietary effect was observed (two-way RM ANOVA, treatment, p = 0.10).

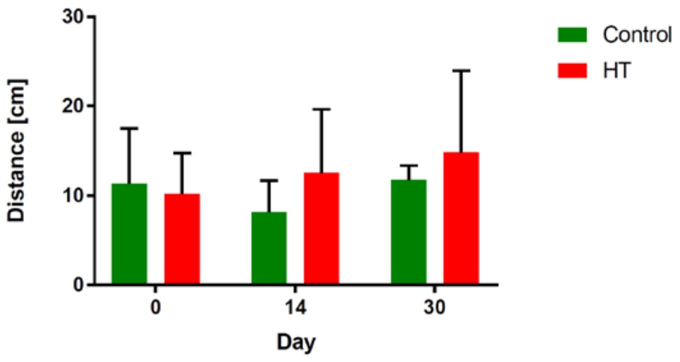

Figure S11. Hydroxytyrosol does not enhance coordination after ischemia. No dietary effect on coordination and balance was found by the rotarod test. Values represent mean ± sd. Control: n= 8, HT: n=8. Statistical analysis was carried out with 2-way RM ANOVA for multiple comparison followed by Holm Sidak's post hoc test for multiple comparisons (*p < 0.05).

Behavioural test - Pole test

The pole test is a general test to assess motor functions.

Baseline levels of velocity did not differ between groups (control: 5.20 ± 0.67 cm/s, HT: 5.37 ± 0.36 cm/s). Velocity did not change over time in both experimental groups. No dietary effect was observed (two-way RM ANOVA, $p > 0.05$).

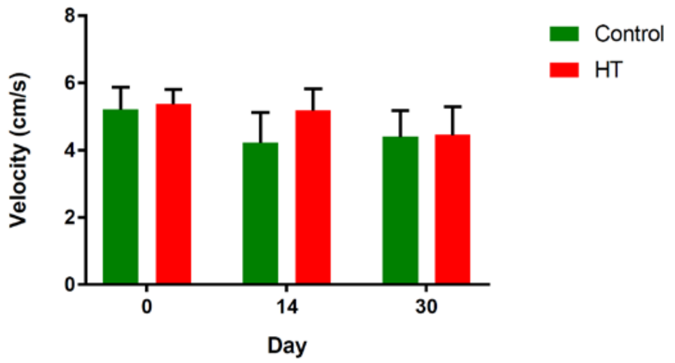

Figure S12. No therapy effect was observed on general motor skills after stroke. No dietary effect on motor skills was observed in both experimental groups. Values represent mean ± sd. Control: n= 8, HT: n=8. Statistical analysis was carried out with 2-way RM ANOVA for multiple comparison followed by Holm Sidak's post hoc test for multiple comparisons (*$p < 0.05$).
